# SRC-2-mediated coactivation of anti-tumorigenic target genes suppresses MYC-induced liver cancer

**DOI:** 10.1371/journal.pgen.1006650

**Published:** 2017-03-08

**Authors:** Shruthy Suresh, Deniz Durakoglugil, Xiaorong Zhou, Bokai Zhu, Sarah A. Comerford, Chao Xing, Xian-Jin Xie, Brian York, Kathryn A. O’Donnell

**Affiliations:** 1 Department of Molecular Biology, UT Southwestern Medical Center, Dallas, TX, United States of America; 2 Department of Immunology, Nantong University School of Medicine, Nantong, China; 3 Department of Molecular and Cellular Biology, Baylor College of Medicine, Houston, TX, United States of America; 4 Department of Molecular Genetics, UT Southwestern Medical Center, Dallas, TX, United States of America; 5 Department of Clinical Sciences, UT Southwestern Medical Center, Dallas, TX, United States of America; 6 McDermott Center for Human Growth and Development, UT Southwestern Medical Center, Dallas, TX, United States of America; 7 Harold C. Simmons Comprehensive Cancer Center, UT Southwestern Medical Center, Dallas, TX, United States of America; 8 Dan L. Duncan Cancer Center, Baylor College of Medicine, Houston, TX, United States of America; Seattle Children's Research Institute, UNITED STATES

## Abstract

Hepatocellular carcinoma (HCC) is the fifth most common solid tumor in the world and the third leading cause of cancer-associated deaths. A *Sleeping Beauty*-mediated transposon mutagenesis screen previously identified mutations that cooperate with MYC to accelerate liver tumorigenesis. This revealed a tumor suppressor role for *Steroid Receptor Coactivator 2*/*Nuclear Receptor Coactivator 2* (*Src-2*/*Ncoa2*) in liver cancer. In contrast, SRC-2 promotes survival and metastasis in prostate cancer cells, suggesting a tissue-specific and context-dependent role for SRC-2 in tumorigenesis. To determine if genetic loss of SRC-2 is sufficient to accelerate MYC-mediated liver tumorigenesis, we bred *Src-2*^*-/-*^ mice with a MYC-induced liver tumor model and observed a significant increase in liver tumor burden. RNA sequencing of liver tumors and *in vivo* chromatin immunoprecipitation assays revealed a set of direct target genes that are bound by SRC-2 and exhibit downregulated expression in *Src-2*^*-/-*^ liver tumors. We demonstrate that activation of *SHP (Small Heterodimer Partner)*, *DKK4* (*Dickkopf-4)*, and *CADM4 (Cell Adhesion Molecule 4)* by SRC-2 suppresses tumorigenesis *in vitro* and *in vivo*. These studies suggest that SRC-2 may exhibit oncogenic or tumor suppressor activity depending on the target genes and nuclear receptors that are expressed in distinct tissues and illuminate the mechanisms of tumor suppression by SRC-2 in liver.

## Author summary

Liver cancer is the third leading cause of cancer-associated deaths worldwide with limited responses to targeted therapies. An unbiased forward genetic screen previously revealed a tumor suppressor role for the *Steroid Receptor Coactivator 2* (*Src-2*) in liver cancer driven by the *MYC* oncogene. Yet, SRC-2 has been shown to promote survival and metastasis in prostate cancer cells, suggesting a tissue-specific and context-dependent role for SRC-2 in tumorigenesis. Through the use of mice lacking SRC-2, we provide unequivocal evidence that this protein restrains MYC-induced liver tumorigenesis, and we have begun to identify key downstream SRC-2 target genes that mediate this effect. This work provides important new insights into the mechanism of tumor suppression by SRC-2 in MYC-induced liver cancer. Our study also suggests that SRC-2 may exhibit oncogenic or tumor suppressor activity depending on the target genes and nuclear receptors that are expressed in distinct tissues.

## Introduction

Hepatocellular carcinoma (HCC) is the fifth most common solid tumor and the third leading cause of cancer-related deaths, resulting in approximately 700,000 deaths per year worldwide [1]. Liver tumorigenesis occurs in settings of chronic inflammation, cirrhosis, or glycogen storage disease [2, 3]. Previous studies have described genomic alterations in human HCC, with recurrent loss of the *TP53* and *RB* tumor suppressor genes, and amplification or overexpression of the *MYC* oncogene in 40–60% of HCCs [4–6]. Despite this wealth of data, the critical genes and pathways that contribute to HCC development are incompletely understood. A better understanding of the mechanisms underlying HCC initiation and progression may accelerate the development of novel therapeutic strategies.

Complementary to large-scale genome sequencing studies, forward genetic mutagenesis screens in mice provide an unbiased approach to study the significance of gene mutations in tumorigenesis [7–12]. Previously, we utilized the *Sleeping Beauty (SB)* DNA transposon system to identify mutations that cooperate with *MYC* to accelerate liver tumorigenesis in mice. This led to the identification of *Steroid Receptor Coactivator 2* (*SRC-2*, also known as *NCOA2*, *TIF2*, *GRIP1*) as a novel gene that functions to restrain MYC-induced liver cancer [13]. *SRC-2* encodes a potent transcriptional coactivator that cooperates with nuclear receptors (NRs) to control multiple physiological processes including glucose homeostasis, energy metabolism, and reproduction [14–22]. Mice with whole-body or liver-specific deletion of *Src-2* develop glycogen storage disease Type 1 (Von Gierke’s disease), and exhibit decreased expression of the SRC-2 target *Glucose 6 phosphatase* (*G6pc*) [15]. Moreover, a significant fraction of patients with Von Gierke’s disease develop hepatic adenomas and are susceptible to developing HCC [23]. Several lines of evidence from our previous study supported a cell-autonomous tumor suppressor role for SRC-2 in liver tumorigenesis [13]. First, recurrent transposon insertions in *SB-*induced liver tumors resulted in decreased mRNA expression of *Src-2* and one of its characterized targets *G6pc*. Second, inhibition of *Src-2* using shRNAs promoted tumor formation by mouse hepatoblasts in immunocompromised mice. Third, deletion of *Src-2* predisposed mice to diethylnitrosamine (DEN)-induced liver tumorigenesis. Finally, we observed decreased expression of *SRC-2* (*NCOA2*) in human HCC samples. Consistent with these findings, depletion of *SRC-2* in human breast cancer cells stimulated cell proliferation by modulating estrogen-regulated genes [24]. Nevertheless, multiple observations suggest that further functional studies of SRC-2 are needed to establish whether this protein is a *bona fide* tumor suppressor in liver cancer. For example, copy number gains of *SRC-2* are frequent in liver cancer [25, 26], although this is likely due to the proximity of this gene to the *MYC* gene on chromosome 8q. Furthermore, a recent study demonstrated that SRC-2 promotes lipogenesis and enhanced cell survival and metastasis in prostate cancer [27], suggesting a tissue-specific and context-dependent role for SRC-2 in tumorigenesis.

To definitively test the tumor suppressor activity of SRC-2 in MYC-mediated liver tumorigenesis *in vivo* and to further investigate the mechanism(s) through which this coactivator inhibits liver tumorigenesis, we examined the consequences of genetic deletion of *Src-2* in a MYC-induced liver cancer model. Indeed, liver tumor burden was significantly increased in *Src-2*^*-/-*^ mice. RNA sequencing (RNAseq) and *in vivo* chromatin immunoprecipitation assays revealed a set of direct SRC-2 target genes in liver. Inhibition of SRC-2 or select SRC-2 target genes accelerated proliferation of human liver cancer cells *in vitro* and tumorigenesis *in vivo*, while overexpression of SRC-2 targets, or SRC-2 itself, resulted in tumor suppressive effects. These findings provide important new insights into the mechanism of tumor suppression by SRC-2 in MYC-induced liver cancer.

## Results

### Deletion of *Src-2* accelerates MYC-mediated liver tumorigenesis

**Fig 1:**
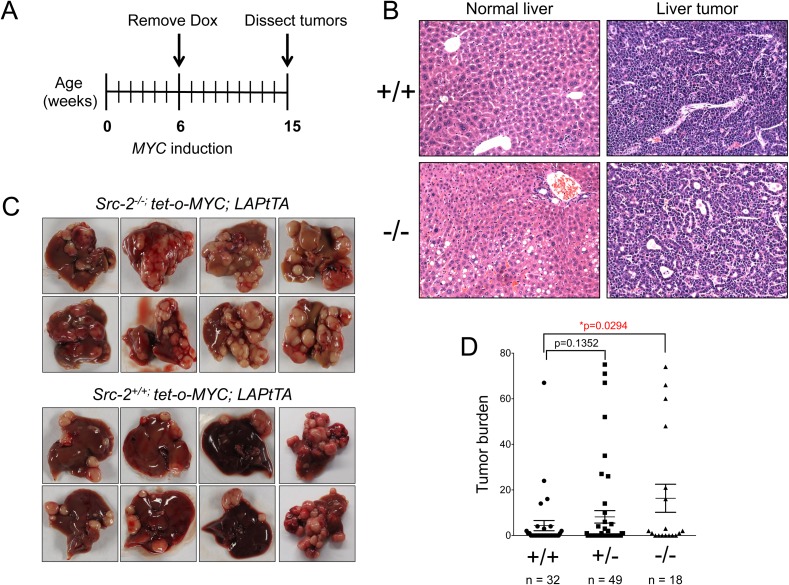
Acceleration of liver tumorigenesis in *Src-2*^-/-^; tet-o-*MYC*; LAPtTA mice. (A) Timeline of MYC induction. Dox, doxycycline. (B) Hematoxylin and eosin (H&E) staining of normal liver and liver tumors from *Src-2*^-/-^ and *Src-2*^+/+^ mice. (C) Representative images of multifocal liver tumors from *Src-2*^-/-^ and *Src-2*^+/+^ animals overexpressing MYC in liver. (D) Quantification of tumor burden from animals that formed liver tumors at time of dissection (15 wk). n = 32 for animals with wildtype (+/+) *Src-2*, n = 49 for *Src-2* heterozygotes (+/-) and n = 18 for *Src-2* knockout mice (-/-); *Src-2* (+/+) vs. (-/-) mice,*p = 0.0294, Wilcoxon rank sum test.

To determine whether SRC-2 suppresses MYC-mediated liver cancer, we employed a mouse model of MYC-induced liver cancer previously utilized in a *SB* mutagenesis screen [28]. Mice harboring a *MYC* transgene under the control of a doxycycline-regulatable promoter (*tet-o-MYC*) were crossed with mice expressing tet-transactivator protein (tTA) driven by the liver-activator protein (LAP) promoter. Removal of doxycycline leads to *MYC* induction in the liver and development of tumors that resemble human hepatocellular cancer. We bred this model to *Src-2*^*+/-*^ mice and generated *tet-o-MYC; LAPtTA* animals harboring wild type, heterozygous, or homozygous null alleles of *Src-2* (**S1A Fig**) [29]. Loss of SRC-2 was confirmed by western blotting with tumor lysates from *Src-2*^*+/+*^ and *Src-2*^*-/-*^ animals (**S1B Fig**)**.** Doxycycline was withdrawn at 6 weeks, and mice were monitored for early-developing tumors (**Fig 1A**). All animals were euthanized and dissected at 15 weeks of age (9 weeks after MYC induction). Histologic analysis confirmed that tumors arising in these animals resembled human hepatocellular cancer (**Fig 1B**) and, consistent with prior reports, *Src-2*^*-/-*^ mice exhibited an accumulation of glycogen and lipid droplets in non-neoplastic hepatocytes and in liver tumors (**S2 Fig**) [15]. Notably, *Src-2*^*-/-*^ mice exhibited a significant enhancement of liver tumor burden compared to *Src-2*^*+/+*^ animals (**Fig 1C and 1D,** p<0.0295). Therefore, genetic inactivation of *Src-2* is sufficient to accelerate MYC-mediated liver tumorigenesis.

### Identification of direct SRC-2-regulated transcripts in MYC-induced liver tumors

**Fig 2:**
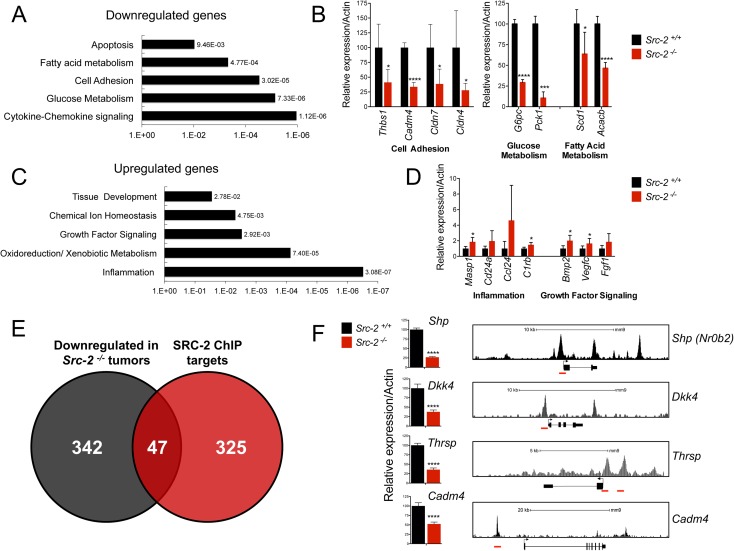
Identification of direct SRC-2 targets in MYC-driven liver tumors. (A) DAVID gene ontology analysis of downregulated genes in *Src-2*^-/-^ liver tumors. Individual p-values of enrichment are depicted next to each biological process. (B) Real-time PCR quantification of cell adhesion, glucose metabolism, and fatty acid metabolism genes in *Src-2*^-/-^ and *Src-2*^+/+^ liver tumors. Bar graphs represent mRNA expression of genes relative to Actin. Error bars represent SDs from five independent samples per group. * = p<0.05; ** = p<0.01; *** = p<0.001; **** = p<0.0001. (C) DAVID gene ontology analysis of upregulated genes in *Src-2*^-/-^ liver tumors. (D) Real-time PCR quantification of inflammation and growth factor signaling genes in *Src-2*^-/-^ and *Src-2*^+/+^ liver tumors. (E) Overlap of downregulated genes in *Src-2*^-/-^ liver tumors and *in vivo* mouse liver SRC-2 ChIP-Seq targets. (F) Real-time PCR quantification of candidate SRC-2 target genes *Shp*, *Dkk4*, *Thrsp* and *Cadm4* in *Src-2*^-/-^ and *Src-2*^+/+^ liver tumors (left). As in (B) and (D), error bars represent SDs from five independent samples per group. SRC-2 ChIP-Seq peaks upstream of transcriptional start sites of candidate genes (right). SRC-2 binding sites are highlighted in red.

To investigate the mechanisms through which SRC-2 suppresses liver tumorigenesis, we used RNA-Seq to assess global gene expression in liver tumor nodules from *Src-2*^*+/+*^ and *Src-2*^*-/-*^ animals. We identified 865 differentially expressed genes between wild type and knockout tumors. DAVID Gene Ontology analysis identified biological processes enriched in *Src-2*^*-/-*^ liver tumors (**Fig 2A and 2C**). Downregulated genes included regulators of fatty acid and glucose metabolism, and cell adhesion. Upregulated genes included mediators of growth factor signaling and inflammation. Key genes from each of these categories were validated using quantitative real-time PCR (qRT-PCR) (**Fig 2B and 2D**). Thus, *Src-2* may function to restrain HCC by regulating multiple biological pathways relevant to tumorigenesis.

To distinguish direct versus indirect SRC-2 target genes, we overlapped the list of genes that were downregulated in *Src-2*^*-/-*^ liver tumors with genes that were bound by SRC-2 in genome-wide chromatin immunoprecipitation (ChIP) Seq analysis of murine liver [17] (**Fig 2E**). We identified 47 genes that were bound by SRC-2 and downregulated in *Src-2*^*-/-*^ liver tumors (**S1 Table**). To identify clinically relevant candidate genes, we used data from a previously described gene expression analysis of human liver tumors and paired adjacent normal tissue [30] to assess expression of 23 of these genes that were downregulated by at least 2-fold in *Src-2*^*-/-*^ liver tumors and were expressed in the human dataset. Of these, 19/23 genes were downregulated in human HCC samples (**S3 Fig**). We selected four putative downstream targets of SRC-2 for further study: *Small Heterodimer Partner (Shp)*, *Dickopff 4 (Dkk4)*, *Cell Adhesion Molecule 4 (Cadm4)*, and *Thyroid hormone responsive (Thrsp)*. These genes were selected because they were downregulated in *Src-2*^*-/-*^ tumors (our RNA-Seq analysis) and in human HCCs, they harbored mutations in human cancers (**S2 Table, S3 Table**), and they were directly bound by SRC-2. Indeed, we confirmed using qRT-PCR that expression of three out of four of these genes (*Shp*, *Dkk4*, and *Cadm4*) was significantly downregulated in an independent set of *Src-2*^*-/-*^ liver tumors (**S4 Fig**), and identified SRC-2 ChIP-seq peaks in the proximal promoter and/or enhancer regions of each gene (**Fig 2F**). Although *Thrsp* was not significantly downregulated in the independent tumors, it was downregulated in a cohort of 91 HCC tumors relative to paired normal adjacent tissue [30] and we therefore included it in selected functional studies.

In our ChIP-Seq analysis, we also found that SRC-2 bound to the proximal promoter of *Vegfc*, *Fgf1*, and *Masp1*, and that mRNA expression was upregulated in *Src-2*^*-/-*^ liver tumors (**S5 Fig**). *Vegfc* and *Fgf1* encode growth factors that promote cell growth and survival [31, 32]. *Masp1* is a key component of the complement cascade, which has also been implicated in promoting tumorigenesis [33, 34]. Although activation of gene targets is thought to serve as the primary function of this nuclear receptor coactivator, SRC-2 was previously reported to cooperate with NRs including Glucocorticoid Receptor and Estrogen Receptor to mediate transcriptional repression [35, 36]. Therefore, we speculate that SRC-2 might also repress downstream target genes that promote growth and proliferation. Future studies are warranted to assess SRC-2-mediated gene repression in the context of liver tumorigenesis.

### SRC-2 targets *SHP*, *DKK4*, and *CADM4* exhibit tumor suppressor activity in human HCC cells

To functionally validate SRC-2 target genes as putative tumor suppressors, we next performed loss-of-function experiments in human HCC cells. HepG2 and Huh7 were chosen for these studies since these cell lines are widely used for functional analysis of genes in HCC and they express MYC at levels comparable to liver tumors in *Src-2*^*-/-*^*; tet-o-MYC; LAPtTA* mice (**S6 Fig**). *DKK4* and *CADM4* were expressed at high levels in HepG2 cells, and *SHP* was highly expressed in Huh7 cells, allowing examination of the consequences of their inhibition in either of these cell lines. *THRSP* was not expressed in Huh7 or HepG2 cells, precluding analysis of *THRSP* loss of function in these cells.

**Fig 3:**
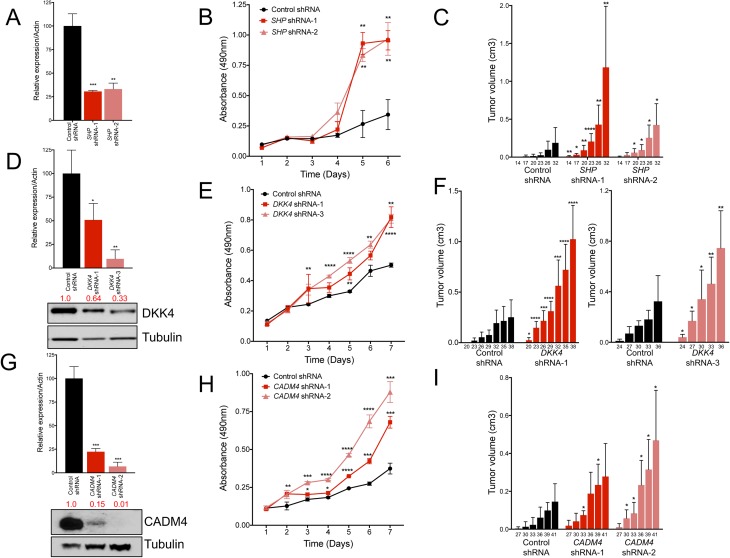
Inhibition of *SHP*, *DKK4*, and *CADM4* accelerate HCC cell proliferation *in vitro* and tumor growth *in vivo*. (A) Real-time PCR quantification of *SHP* expression in Huh7 cells after inhibition with two independent shRNAs. Bars graphs represent mRNA expression of *SHP* normalized to *ACTIN* and error bars represent SDs from triplicate measurements. (B) MTS proliferation assay measuring proliferation of *SHP* shRNA and control shRNA cells over time. (C) Quantification of tumor volumes in nude mice injected with Huh7 cells with *SHP* shRNAs or control shRNA. (D) Top, real-time PCR quantification of *DKK4* expression in HepG2 cells after inhibition with two independent shRNAs. Bar graphs represent *DKK4* mRNA expression normalized to *ACTIN* and error bars represent SDs from triplicate measurements. Bottom, western blot with quantification of DKK4 protein levels and normalized to Tubulin. (E) MTS proliferation assay measuring the proliferation of *DKK4* shRNA and control shRNA cells with over time. (F) Quantification of tumor volumes in nude mice injected with HepG2 cells with *DKK4* shRNAs or control shRNA. (G) Real-time PCR quantification and western blot analysis of *CADM4* mRNA and protein in HepG2 cells after inhibition with two independent shRNAs. (H) MTS assay measuring proliferation of *CADM4* shRNA and control cells over time. (I) Quantification of tumor volumes in nude mice injected with HepG2 cells with *CADM4* shRNAs or control shRNA. Bar graphs (C), (F), and (I) represent mean tumor volumes. For this and all subsequent xenograft experiments, the numbers below each bar represent the time (in days) after subcutaneous injection of cells into nude mice. Error bars in real-time quantitation and proliferation assays represent SDs from triplicate measurements. Error bars in xenograft experiments represent SDs from a total of ten subcutaneous injections (n = 5 mice) per shRNA tested. A student’s t-test was performed to determine statistical significance. * = p<0.05; ** = p<0.01; *** = p<0.001; **** = p<0.0001.

*SHP* encodes an orphan nuclear receptor that lacks a conserved DNA binding domain and physically interacts with nuclear receptors and transcriptional factors to facilitate transcriptional repression [37, 38]. In the liver, SHP transcriptionally represses *CYP7A1* to regulate bile acid biosynthesis. Loss of *Shp* in mice results in abnormal accumulation of bile acids and liver tumor development [39, 40]. To determine whether *SHP* inhibition promotes proliferation and tumorigenesis in human cells, we utilized shRNAs to suppress *SHP* in Huh7 cells. qRT-PCR confirmed inhibition of *SHP* mRNA using two independent shRNAs (**Fig 3A**). Cells with stable inhibition of *SHP* grew significantly faster than control cells (**Fig 3B**). Moreover, *SHP* depletion accelerated tumor formation of Huh7 cells in immunocompromised mice (**Fig 3C**). Although we detected an increase in *Cyp7a1* in *Src*^*-/-*^ tumors (**S7A Fig**), *CYP7A1* was not expressed in human HCC cells. Taken together, our data provide evidence that SHP is a downstream target of SRC-2 that inhibits liver tumorigenesis.

A previous study demonstrated that *SHP* suppressed proliferation by transcriptionally repressing *Cyclin D1 (Ccnd1)* expression and that *Shp*^*-/-*^ liver tumors exhibited increased *Ccnd1* expression [40]. However, it was also reported that CCND1 levels were unaffected in livers of mice overexpressing SHP [41]. Notably, we failed to observe a significant change in *CCND1* mRNA or protein in Huh7 cells after *SHP* knockdown (**S7B and S7C Fig**). Similarly, we failed to detect a difference in *CCND1* mRNA in Huh7 xenograft tumors lacking *SHP* (**S7D Fig)**. These findings suggest that in addition to its known effects on bile acid homeostasis, SHP suppresses liver tumorigenesis by regulating tumor cell proliferation through a mechanism that is independent of CCND1.

We next utilized shRNAs to inhibit expression of *DKK4* and *CADM4* in HepG2 cells. *DKK4* belongs to the Dickopff (DKK) family of secreted glycoproteins and negatively regulates Wnt signaling [42]. qRT-PCR and western blotting confirmed a reduction in mRNA and protein, respectively (**Fig 3D**). *DKK4* shRNA-1 and shRNA-3 cells grew significantly faster than control cells (**Fig 3E**). Moreover, depletion of DKK4 enhanced tumorigenesis *in vivo* (**Fig 3F**). Similarly, inhibition of *CADM4*, which encodes a cell adhesion molecule that belongs to the immunoglobulin superfamily [43], significantly increased cell proliferation and tumorigenesis *in vivo* (**Fig 3G–3I**). Thus, multiple SRC-2 target genes, including *SHP*, *CADM4*, and *DKK4*, exhibit tumor suppressor activity in human HCC cells.

### Overexpression of SRC-2 or its targets suppresses tumor formation

**Fig 4:**
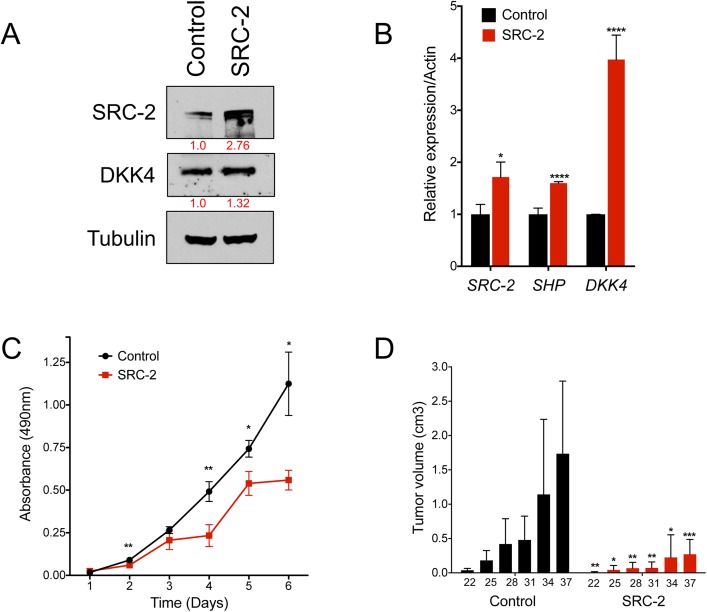
Overexpression of SRC-2 upregulates candidate gene expression and reduces HCC cell tumor formation *in vivo*. (A) Western blot demonstrating expression of SRC-2 and DKK4 levels in Huh7 cells. Cells were infected with pLJM1 lentiviruses expressing eGFP (as a control) or SRC-2. (B) Real-time PCR quantification of *SRC-2*, *SHP* and *DKK4* expression in Huh7 cells expressing eGFP or SRC-2. (C) MTS assay measuring proliferation of cells overexpressing SRC-2. Error bars in (B) and (C) represent SDs from triplicate measurements. Student’s unpaired t-test was used to evaluate statistical significance. * = p<0.05; ** = p<0.01; **** = p<0.0001. (D) Quantification of tumor volumes of nude mice injected subcutaneously with Huh7 cells overexpressing SRC-2 or control eGFP. Bar graphs represent mean tumor volumes. Error bars represent SDs from a total of ten subcutaneous injections (n = 5 mice) per experimental group tested. Student’s unpaired t-test was used to evaluate statistical significance * = p<0.05; ** = p<0.01; *** = p<0.001.

We next determined whether SRC-2 overexpression is sufficient to suppress tumorigenesis in human liver cancer cells. Huh7 cells were infected with an *SRC-2*-expressing or an eGFP control lentivirus, and overexpression of SRC-2 was confirmed by quantitative RT-PCR and western blotting **(Fig 4A and 4B)**. Upregulation of SRC-2 and its target *SHP* (**Fig 4B**) were associated with a concomitant decrease in cell proliferation and tumorigenesis in immunocompromised mice **(Fig 4C and 4D)**. Thus, as in mouse, SRC-2 restrains liver tumorigenesis in human HCC cells. Notably, although DKK4 transcript levels increased by 4-fold upon SRC-2 overexpression, DKK4 protein levels were only modestly affected, suggesting the existence of post-transcriptional mechanisms that control DKK4 expression independently of SRC-2 in these cells.

**Fig 5:**
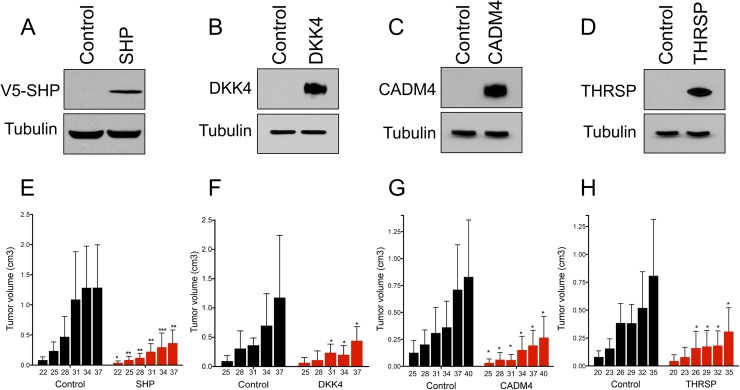
Overexpression of *SHP*, *DKK4*, *CADM4* and *THRSP* reduce tumor formation of Huh7 cells *in vivo*. (A-D) Western blot demonstrating overexpression of SRC-2 targets SHP, DKK4, CADM4 and THRSP in Huh7 cells. A V5 antibody is used for detection of SHP overexpression. (E-H) Quantification of tumor volumes of nude mice injected subcutaneously with Huh7 cells overexpressing SHP, DKK4, CADM4 and THRSP, respectively. Bar graphs represent mean tumor volumes. Error bars represent SDs from a total of ten subcutaneous injections (n = 5 mice) per experimental group tested. Student’s unpaired t-test was used to evaluate statistical significance * = p<0.05, ** = p<0.01, *** = p<0.001.

To validate the ability of SRC-2 targets to suppress tumorigenesis, we next overexpressed individual target genes in human HCC cells using lentivirus and assessed tumor development *in vivo*. Complementary to the loss-of-function experiments (**Fig 3**), *SHP*, *DKK4*, or *CADM4* overexpression significantly reduced tumor formation in immunocompromised mice (**Fig 5A–5C and 5E–5G)**. We also observed reduced tumor formation upon enforced expression of *THRSP* (**Fig 5D and 5H**), which encodes an acidic protein that responds robustly to thyroid hormone stimulus [44] that has not been previously linked to liver cancer.

### SHP and CADM4 suppress enhanced tumor burden resulting from SRC-2 inhibition

**Fig 6:**
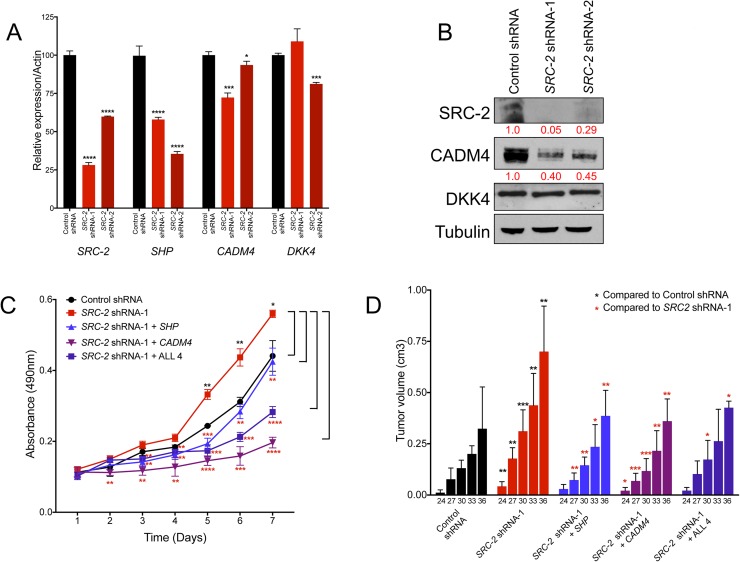
SHP and CADM4 rescue enhanced tumor burden upon SRC-2 inhibition. (A) Real-time PCR quantification of *SRC-2*, *SHP*, *CADM4*, and *DKK4* expression in HepG2 cells after inhibition of *SRC-2* with two independent shRNAs. Bar graphs represent mRNA expression of the labeled transcript normalized to *ACTIN* and error bars represent SDs from triplicate measurements. (B) Western blot analysis of SRC-2 and its targets CADM4 and DKK4 in HepG2 cells after SRC-2 inhibition with two independent shRNAs. Numbers in red represent quantification of protein levels relative to the control shRNA sample. (C) MTS assay measuring proliferation of HepG2 cells with control shRNA, *SRC-2* shRNA-1, or *SRC-2* shRNA-1 with overexpression of *SHP* or *CADM4* alone, or in combination with *THRSP* and *DKK4* (labeled as ALL 4). (D) Quantification of tumor volumes in nude mice injected with HepG2 cells as described in (C). Bars represent mean tumor volumes. Error bars in real-time quantitation and proliferation assays represent SDs from triplicate measurements. Error bars in xenograft experiments represent SDs from a total of ten subcutaneous injections (n = 5 mice) per shRNA tested. A student’s t-test was performed to determine statistical significance. * = p<0.05; ** = p<0.01; *** = p<0.001; **** = p<0.0001. Black asterisks represent comparisons to the control shRNA. Red asterisks represent comparisons to SRC-2 shRNA-1.

We previously demonstrated that inhibition of *Src-2* promoted tumor formation of murine hepatoblasts in immunocompromised mice [13]. To expand these findings to human liver cancer cells, we performed *SRC-2* loss of function studies in HepG2 and Huh7 cells. As expected, *SRC-2* inhibition in HepG2 cells resulted in decreased expression of SRC-2 and its targets *SHP* and *CADM4*, and significantly increased cell proliferation and tumorigenesis *in vivo* (**Fig 6A–6D**). Similarly, inhibition of SRC-2 in Huh 7 cells resulted in decreased *SHP* and *DKK4* expression, and a concomitant increase in cell proliferation (**S8A and S8B Fig**). We next sought to determine whether any of the SRC-2 targets alone or in combination were sufficient to rescue the enhanced cell proliferation and tumor burden resulting from SRC-2 knockdown. Rescue experiments were performed in HepG2 cells because three of the four putative SRC-2 target genes (*SHP*, *CADM4*, and *DKK4*) were expressed in these cells. Indeed, enforced expression of SHP, CADM4, DKK4, and THSRP in combination significantly reduced proliferation and tumor burden (**Fig 6C and 6D, S9A Fig**). Moreover, individual overexpression of CADM4 and SHP were sufficient to suppress the increase in cell proliferation and tumorigenesis of SRC-2 knockdown cells (**Fig 6C and 6D, S9A Fig**). In contrast, overexpression of either DKK4 or THRSP alone significantly impacted rates of cell proliferation but not tumor burden (**S9A–S9C Fig**). These data provide convincing evidence that *SHP* and *CADM4* function as important anti-tumorigenic SRC-2 target genes in human liver cancer cells. Our data also suggest that DKK4 and THRSP may not be targets of SRC-2 in HepG2 cells, and thus may be dysregulated in liver cancer cells through additional SRC-2-independent mechanisms.

### Analysis of nuclear receptor binding motifs associated with SRC-2-target gene promoters

Finally, we sought to identify the putative nuclear receptors that cooperate with SRC-2 to activate transcription of target gene expression and suppress proliferation and tumorigenesis. We screened the promoter regions of *DKK4*, *THRSP*, *CADM4*, and *SHP* for nuclear receptor binding motifs using NHRscan, a computational predictor of nuclear hormone receptor binding sites [45]. We then assessed whether the NR binding motifs overlapped with SRC-2 ChIP-Seq peaks identified in this study. This analysis revealed that the promoter regions of *Dkk4* and *Thrsp* both contained Thyroid Receptor (TR) binding motifs, denoted as Everted Repeat 6 (ER6) (**S10A and S10B Fig**) [46]. Recently, ChIP-Seq analysis identified a TR peak upstream of the *Thrsp* promoter, although TR binding to *Dkk4* was not verified in this study [47]. However, TR is known to inhibit liver tumorigenesis through transcriptional activation of *DKK4* [48]. NHRscan analysis also uncovered several Direct Repeats (DR) that overlap with SRC-2 binding regions upstream of *CADM4* (**S10C Fig**). Previous studies showed that RAR heterodimerizes with RXRA and preferentially binds to DR-rich regions in the genome [49, 50]. Finally, NR binding motif analysis revealed that the *SHP* promoter harbors HNF4A and FXR binding motifs overlapping with SRC-2 ChIP-Seq peaks (**S10D Fig**). Indeed, genome-wide ChIP-Seq analysis in mouse liver identified a FXR peak that overlaps with the SRC-2 ChIP Seq peak [51].

To determine whether SRC-2 cooperates with FXR in activating SHP expression in human liver cancer cells, we performed transactivation assays with a luciferase reporter construct harboring the proximal promoter of *SHP*, and a truncated reporter construct harboring a deletion that encompasses the FXR binding site. FXR was previously shown to activate the human *SHP* (NR0B2) promoter [52]. FXR was expressed in Huh7 cells infected with an eGFP control or SRC-2 lentivirus. Overexpression of SRC-2 and FXR increased *SHP* reporter activity by approximately 9-fold in Huh7 cells compared to cells expressing FXR alone (**S11A–S11C Fig**). Interestingly, while the truncated reporter construct was significantly less active, it was also measurably stimulated by SRC-2 expression. These findings suggest that SRC-2 can interact with other factors that transactivate the SHP promoter. These data provide additional evidence that SRC-2 directly induces SHP expression. Future studies are warranted to dissect additional SRC-nuclear receptor interactions in liver cancer and in different tumorigenic contexts.

## Discussion

Recently, large-scale studies have identified multiple types of recurrent genomic alterations of *SRC-2* in human HCC, including missense mutations and amplifications [25, 26]. Notably, *SRC-2* and *MYC* are both located on the short arm of chromosome 8. *MYC* is amplified in 40–60% of human HCCs and a number of studies have previously documented 8q gains in a significant fraction of liver cancers [53–55]. Thus, it is possible that SRC-2 copy number gains may occur simply due to a passenger effect associated with *MYC* amplification and may not functionally contribute to tumorigenesis. In support of this concept, Kaplan-Meier analysis revealed that survival of HCC patients with SRC-2 amplification or mRNA upregulation was not significantly different than survival of patients lacking these alterations (**S12 Fig**). In contrast, we previously showed that low expression of *SRC-2* in tumors is strongly associated with poor survival in HCC patients [13, 56] and HCC patients harboring *SRC-2* missense mutations similarly exhibit poorer overall survival (**S12 Fig**). Taken together, these studies point to a tumor suppressor role for SRC-2 in HCC. Nevertheless, in light of recent evidence indicating that SRC-2 has oncogenic activity in prostate cancer [27], a direct demonstration of the tumor suppressor activity of SRC-2 in liver cancer, and a better understanding of the underlying mechanisms, would provide important insight into the role of SRC-2 in HCC. Through the use of *Src-2*^*-/-*^ mice, we have now provided unequivocal evidence that this protein restrains *MYC*-mediated liver tumorigenesis *in vivo* and we have begun to identify key downstream SRC-2 target genes that mediate this effect.

The orphan nuclear receptor *SHP* represents one such direct SRC-2 target gene with strong anti-tumorigenic activity. SHP has been extensively studied for its role in liver bile acid homeostasis and as a transcriptional repressor of other NRs. Mice lacking *Shp* accumulate bile acids due to de-repression of the SHP target *Cyp7a1* and develop HCC [39, 40]. *SHP* is also downregulated in liver cancer and low expression of *SHP* is associated with poor survival of HCC patients [57]. Accordingly, our data demonstrate that *SHP* inhibition accelerates tumor formation by human HCC cells in mice. Although we detected an increase in *Cyp7a1* in *Src*^*-/-*^ tumors, we did not detect expression of *CYP7A1* in human HCC cells, nor did we detect a difference in expression of another putative SHP target, *Ccnd1* (**S7 Fig**). These data suggest that *SHP* represses hepatic tumorigenesis through mechanisms that are independent of these genes. Importantly, overexpression of SHP alone was sufficient to reverse the tumor enhancing effect of SRC-2 knockdown in HepG2 cells (**Fig 6C and 6D, S9A Fig**). In light of these findings, future studies are warranted further characterize SHP targets that control proliferation and metabolism in liver cancer and other tumor types. It will also be worthwhile to investigate whether treatment with NR agonists that are known to induce SHP expression may inhibit liver tumorigenesis. These studies may impact our understanding and treatment of additional types of cancers as *SHP* was recently found to be downregulated in lung tumors and low expression was associated with poor survival of stage I non-small cell lung cancer patients [58].

*DKK4* was also identified as a novel anti-tumorigenic SRC-2 target gene in this study. *DKK4* encodes a secreted glycoprotein that competes with Wnt ligand binding to LRP5/6 to attenuate canonical Wnt signaling [59]. Dysregulation of the Wnt pathway is a key molecular lesion in liver cancer. More than 60% of liver tumors exhibit an accumulation of β–catenin, a hallmark of activated Wnt signaling. Recent findings demonstrated that DKK4 overexpression suppressed migration, invasion, and tumor formation of human hepatoma cells in mice [42, 48]. Consistent with these data, our findings revealed that *DKK4* suppresses tumorigenesis of human HCC cells *in vivo*, whereas shRNA-mediated inhibition of *DKK4* accelerated tumorigenesis (**Fig 3D–3F, Fig 5B and 5F**). Collectively, these data suggest that *DKK4* may be an important downstream component of the SRC-2-regulated gene expression network that inhibits liver tumorigenesis and uncovers functional antagonism between SRC-2 and the Wnt signaling pathway. However, it is important to note that *DKK4* mRNA and protein levels did not correlate in the SRC-2 gain-of-function (**Fig 4**) and loss of function studies (**Fig 6**), and that overexpression of DKK4 alone was insufficient to rescue the tumor burden of SRC-2 knockdown in HepG2 cells (**S9 Fig**). Thus, there are likely additional mechanisms independent of SRC-2 that control DKK4 expression in liver cancer cells.

We also demonstrated that two additional genes without a prior known role in liver cancer, *CADM4* and *THRSP*, have strong anti-tumorigenic activity in this tumor type. Consistent with these results, expression of *CADM4*, which encodes a member of the immunoglobulin superfamily of proteins, is reduced in multiple tumor types and suppresses tumor formation of prostate, renal and colon cancer cells in immunocompromised mice [60, 61]. Moreover, overexpression of CADM4 was sufficient to reverse tumor acceleration by SRC-2 knockdown in HepG2 cells (**Fig 6C and 6D, S9A Fig**). *THRSP* encodes a key modulator of lipogenesis and is expressed in lipogenic tissues such as liver, breast, and adipose tissue [62]. Although *Thrsp* did not exhibit consistent downregulation in *Src*^*-/-*^ tumors, a previous gene expression analysis [30] revealed that *THRSP* was significantly downregulated in a cohort of 91 HCC tumors relative to paired normal adjacent tissue (**S3 Fig**). Moreover, THRSP inhibited growth and induced cell death of human breast cancer cells [63]. Although THRSP was not expressed in either of the human liver cancer cell lines we tested, enforced expression of THRSP significantly reduced tumor burden of Huh7 cells *in vivo* (**Fig 5D and 5H**). These findings set the stage for further study of the roles of CADM4 and THRSP in HCC pathogenesis.

In summary, these results firmly establish the potent anti-tumorigenic activity of SRC-2 in human and mouse liver cancer and begin to dissect the SRC-2-regulated gene expression network that mediates these effects. Furthermore, these studies provide insight into the molecular mechanisms through which this transcriptional coactivator may limit tumorigenesis in some tissues and promote oncogenesis in others. In the prostate, SRC-2 amplification coactivates androgen receptor-mediated gene transcription to promote prostate lipogenesis, tumor progression, and metastasis [27]. In liver, SRC-2 cooperates with multiple nuclear receptors, several of which are documented tumor suppressors, including Thyroid Receptor (TR), Estrogen Receptor (ER), Hepatocyte Nuclear Factor 4 alpha (HNF4A), Retinoid X Receptor alpha (RXRA), Farnesoid X Receptor (FXR), and Retinoic Acid Receptor alpha (RARA) [15, 17–20, 24, 64, 65] to coactivate a distinct program of target genes resulting in tumor suppression. Recently, a small molecule that stimulated SRC transcriptional activity was developed and shown to promote cell death in breast cancer cells [66]. Determining whether small molecule-mediated activation of SRC-2 can attenuate liver tumorigenesis represents an exciting area for future investigation.

## Materials and methods

### Ethics statement

Mice were monitored closely throughout all experimental protocols to minimize discomfort, distress, or pain. Signs of pain and distress include disheveled fur, decreased feeding, significant weight loss (>20% body mass), limited movement, or abnormal gait. If any of these signs were detected, the animal was removed from the study immediately and euthanized. All sacrificed animals were euthanized with CO2. The animals were placed in a clear chamber and 100% CO2 was introduced. Animals were left in the container until clinical death ensured. To ensure death prior to disposal, cervical dislocation was performed while the animal was still under CO2 narcosis. All methods were performed in accordance with the recommendations of the Panel on Euthanasia of the American Veterinary Medical Association and protocols approved by the UT Southwestern Institutional Animal Care and Use Committee (protocol # 2011–0119).

### Cell culture

HepG2 and Huh7 cells were cultured in Dulbecco’s Modified Eagle Medium (GIBCO) supplemented with 10% FBS (Invitrogen) and 1% Penicillin/Streptomycin (Invitrogen). Huh7 and HepG2 cells were a gift from Hao Zhu (UT Southwestern Medical Center).

### Animals

The Institutional Animal Care and Use Committeee (IACUC) of UT Southwestern Medical Center approved all procedures involving mice. *Src-2*^-/-^mice were obtained from Pierre Chambon and maintained on a mixed C57BL/6J and 129sV background [29]. LAPtTA and tet-O-MYC mice were obtained from Dean Felsher and maintained on a FVB/NJ background [28]. Simultaneously, *Src-2*^*+/-*^ mice were bred with *tet-o-MYC* and *LAPtTA* mice to generate *Src-2*^*+/-*^*; tet-o-MYC* and *Src-2*^*+/-*^*; LAPtTA* mice, respectively. In the final cross, *Src-2*^*+/-*^*; LAPtTA* females were bred with *Src-2*^*+/-*^*; tet-o-MYC* males to obtain *tet-o-MYC; LAPtTA* mice with all 3 alleles of *Src-2* (WT, heterozygous, or homozygous null). The *MYC* transgene is on chromosome Y, precluding analysis of females.

### Plasmids

The following plasmids were used: TRC shRNA for SHP (UT Southwestern core facility, Jerry Shay laboratory V2LHS_239330, V2LHS_72556); TRC shRNA constructs for DKK4 (GE Dharmacon RHS4430-200191360 V2LHS_197942 RHS4430-200173366—V2LHS_204025); GIPZ shRNA for CADM4 (GE Dharmacon V3LHS_375253, V3LHS_375254); GIPZ shRNA for SRC-2 (GE Dharmacon V2LHS_199063, V2LHS_357381). pLJM1-EGFP was a gift from David Sabatini (Addgene plasmid # 19319), and pLX304, which also harbors a V5 tag, was a gift from David Root (Addgene plasmid # 25890). A human *SHP* plasmid was a gift from Steven Kliewer (UT Southwestern Medical Center); pCMX-FXR and hSHP-LUC plasmids were a gift from David Mangelsdorf (UT Southwestern Medical Center). A SHPΔ215-569-LUC deletion mutant construct lacking the FXR response element was generated by PCR amplification as previously described [52]. The pHRL-SV40 Renilla reporter plasmid was a gift from Joshua Mendell (UT Southwestern Medical Center).

### Liver tumor analysis

Whole liver was dissected from euthanized mice, washed, and placed in ice-cold PBS. At the time of dissection, we captured images of both the dorsal and ventral sides of the intact liver, and estimated the mean percent tumor burden for each mouse using NIH Image J software. We measured the surface area of the liver tumors and the total surface area (including normal liver and all tumors). For percent tumor burden calculation, we divided the surface area of the liver tumors by the total surface area (including normal liver and tumors) and then multiplied by 100. For histological analysis, tissues were fixed in 10% formalin, embedded in paraffin, and sectioned. Hematoxylin and eosin (H &E) and Periodic acid-Schiff (PAS) staining were performed on normal liver and liver tumor tissues at the Pathology Core, UT Southwestern Medical Center.

### RNA extraction and qRT-PCR analysis

Total RNA was isolated from liver tumors and normal tissues using Trizol (Invitrogen) followed by additional cleanup and DNase digestion using the RNeasy Mini Kit (Qiagen). Total RNA was isolated from cells using only the RNeasy Mini Kit (Qiagen). For qRT-PCR of mRNA, cDNA synthesis was performed with 1 μg RNA for reverse transcription using Superscript III First Strand synthesis kit (Invitrogen). mRNA expression was assessed using quantitative real-time PCR with a 2X SYBR Green Master Mix (R&D Systems). mRNA levels were normalized to β-actin mRNA expression, with gene expression levels measured using a standard curve for each set of primers crossing exon-exon junctions for each gene. All PCR assays were performed in triplicate. PCR primers are shown in S4 Table.

### RNA sequencing and gene ontology analysis

RNA sequencing was performed in the McDermott Center Sequencing Core at UTSW Medical Center. RNA was extracted from *tet-o-MYC; LAPtTA; Src-2*^*+/+*^ and *tet-o-MYC; LAPtTA; Src-2*^*-/-*^ liver tumors. Four μg of total DNAse treated RNA was prepared with the TruSeq Stranded Total RNA LT Sample Prep Kit from Illumina. Poly-A RNA was purified and fragmented before strand specific cDNA synthesis. cDNA was A-tailed and indexed adapters were ligated. Samples were PCR amplified and purified with AmpureXP beads and validated on the Agilent 2100 Bioanalyzer. Samples were quantified by Qubit (Invitrogen) prior to normalization and pooling. Sequencing was performed on an Illumina Hiseq 2500 to generate 51-bp single-end reads. Reads were trimmed to remove low-quality regions in the ends. Trimmed reads were mapped to the mouse genome (mm10) using TopHat v2.0.1227 guided by iGenomes GTF file (https://ccb.jhu.edu/software/tophat/igenomes.shtml). Alignments with mapping quality less than 10 were discarded. Expression abundance estimation and differential expression analysis were carried out using Cufflinks/Cuffdiff (v2.1.1) software. Genes with the nominal p-value cutoff of 0.05 were considered significantly differentially expressed between the *tet-o-MYC; LAPtTA; Src-2*^*+/+*^ and *tet-o-MYC; LAPtTA; Src-2*^*-/-*^ liver tumors if the genes were also downregulated in human HCCs, harbored mutations in human cancers, and were directly bound by SRC-2 (based on ChIP-Seq data in mouse liver).

Gene Ontology analysis was performed using the DAVID Functional Annotation tool (http://david.abcc.ncifcrf.gov/) on differentially expressed genes between the *tet-o-MYC; LAPtTA; Src-2*^*+/+*^ and *tet-o-MYC; LAPtTA; Src-2*^*-/-*^ liver tumors to identify biological processes specifically enriched in the *Src-2*^*-/-*^ group. Biological processes were assessed for statistical significance (p <0.05).

### ChIP-Seq

ChIP-Seq for SRC-2 (at CT4) was performed by Active Motif, Inc. (Carlsbad, CA) as previously described with no additional filtering [17]. Briefly, mouse liver samples were submerged in PBS containing 1% formaldehyde, cut into small (~1 mm^3^) pieces with a razor blade and incubated at room temperature for 15 minutes. Fixation was stopped by the addition of 0.125 M glycine (final concentration). The tissue pieces were then treated with a TissueTearer and finally spun down and washed twice in PBS. Chromatin was isolated by the addition of lysis buffer, followed by disruption with a Dounce homogenizer. Lysates were sonicated and the DNA was sheared to an average length of 300–500 bp. Genomic DNA (Input) was prepared by treating aliquots of chromatin with RNase, Proteinase K and heated for reverse-crosslinking, followed by ethanol precipitation. Pellets were resuspended and the resulting DNA was quantified on a NanoDrop spectrophotometer. An aliquot of chromatin (30 μg) was precleared with protein A agarose beads (Invitrogen). Genomic DNA regions of interest were isolated using 4 μg of antibody. Complexes were washed, eluted from the beads with SDS buffer, and subjected to RNase and proteinase K treatment. Crosslinking was reversed by incubation overnight at 65°C, and ChIP DNA purified by phenol-chloroform extraction and ethanol precipitation. Illumina sequencing libraries were prepared from the ChIP and input DNAs by the standard consecutive enzymatic steps of end-polishing, dA-addition, and adaptor ligation. After a final PCR amplification step, the resulting DNA libraries were quantified and sequenced on Illumina NextSeq 500 (75 nt reads, single-end).

### ChIP-Seq peak calling and data normalization

The sequences identified were mapped to the mouse genome (NCBI37/UCSC mm9) using BOWTIE function in Galaxy. Only the sequences uniquely mapped with no more than 2 mismatches were kept and used as valid reads. PCR duplicates were also removed. Peak calling was carried out by MACS (version 1.4.2 20120305) in Galaxy/Cistrome (options—mfold 10, 30—pvalue 1x10^-5^), on each ChIP-Seq file against the matching input file. To account for the different sequencing depths between samples, the signal files generated from MACS were normalized to sequencing depth [67]. The peak summits were used as the binding site centers, and the normalized signal files were used as the binding strength for further analysis. Assigning peaks to a given gene was performed with the Genomic Regions Enrichment of Association Tool (version 3.0.0) using the basal plus extension setting [68].

### Western blotting

Cells and tissues were lysed in RIPA buffer and then homogenized using a Bioruptor sonicator (Diagenode). Proteins were quantified using the Bicinchoninic Acid (BCA) assay (Thermo Scientific) and subject to separation by using NuPage Bis-Tris gels (Invitrogen) for electrophoresis. The proteins were subsequently transferred to a nitrocellulose membrane. The membranes were blocked for 1 hour at room temperature and subsequently probed with primary antibodies overnight at 4°C. After incubating the membrane with the appropriate secondary antibody conjugated to horseradish peroxidase, protein levels were detected with SuperSignal Dura substrate (Thermo Scientific). Primary antibodies were prepared in 5% Milk or BSA in TBST. Antibodies were purchased from the following sources: SRC-2 (BD Biosciences, 1:250); DKK4 (Abgent, 1:1000); CADM4 (Neuromabs, 1:500); THRSP (Santa-cruz, 1:500); FXR (Santa Cruz, 1:50). SHP overexpression was detected with a V5 antibody (Invitrogen, 1:5000).

### shRNA mediated depletion

Human Embryonic Kidney 293T (HEK 293T, 1x10^8^) cells were co-transfected with pLKO shRNA constructs (TRC, GE Dharmacon), and PAX2, MD2 helper plasmids using Lipofectamine 2000 (Life technologies). Following transfection, the lentiviral supernatant was collected, filtered and supplemented with 8ug/ml hexadimethrene bromide (Sigma). Human HCC cell lines Huh7 and HepG2 (3x10^5^) were infected overnight twice with the viral supernatant and 24h after the second infection transferred into fresh media containing Puromycin (2 μg/ml). Cells were selected in puromycin media for at least 7 days and then harvested for RNA or western blot analysis to assess extent of knockdown.

### Lentiviral overexpression

To overexpress candidate genes in human HCC cells, human ORFs corresponding to each gene were cloned into the PLX304 or PLJM1 lentiviral plasmids. PLJM-eGFP or PLX303-empty constructs were used as negative controls. HEK 293T cells (1x10^8^ cells) were then co-transfected with lentiviral overexpression or control constructs and helper plasmids PAX2 and MD2 using Lipofectamine 2000 (Life Technologies). Following transfection, the lentiviral supernatant was collected, filtered and supplemented with 8 μg/ml hexadimethrene bromide (Sigma). Human Huh7 cells (3x10^5^) were infected overnight twice with the viral supernatant and 24h after the second infection transferred into fresh media containing blasticidin (4 μg/ml) or puromycin (2μg/ml). Control cells and cells overexpressing SRC-2 or SRC-2 target genes were selected in antibiotic-containing media for at least 7 days and then harvested for RNA and western blot analysis to assess overexpression.

### Xenograft assays

Human HCC cells (3–5 x 10^6^) expressing shRNA lentiviruses or lentiviruses overexpressing candidate genes in PBS were injected subcutaneously into both the left and right flanks of 6 week-old immunocompromised athymic nude mice (Charles River, strain 490). Tumor volume was measured using calipers every 3–4 days until the average tumor mass reached 2cm^3^. Tumor volume was calculated using the formula (length x width^2^)/2. A total of five mice were injected per experimental group, corresponding to ten experimental samples per group.

### Cell proliferation assays

To measure *in vitro* proliferation of cells, the CellTiter 96 Aqueous Non-Radioactive Cell Proliferation assay kit (Promega) was used. 1000 cells per well were plated in 96-well plates in triplicate overnight. The MTS/PMS agent was added to the media according to the manufacturer’s protocol and incubated at 37°C for 1.5 hours. Absorbance was then measured at 490 nm every 24 hours for 6–7 days. All experiments were performed in triplicate and performed at least two times.

### Nuclear hormone receptor (NR) binding site analysis

To predict NRs that interact with SRC-2, promoter regions (spanning 10kb on either side) of candidate SRC-2 target genes were screened for NR binding motifs using the NHR scan tool (http://www.cisreg.ca/cgi-bin/NHR-scan/nhr_scan.cgi). SRC-2 binding regions in candidate genes were overlapped with predicted NR binding motifs to predict potential SRC-2/NR interactions.

### Dual luciferase assays

5 x10^4^ Huh7 cells expressing an eGFP control or SRC-2 lentivirus were seeded per well in 12-well plates in triplicate. Cells were transfected 24 hours later using Fugene HD (Promega) with 20 ng FXR plasmid, 80 ng SHP-LUC or SHP^Δ215-569^-LUC reporter plasmids, 1 ng Renilla control reporter plasmid and 199ng pUC19 plasmid to give a total of 300 ng DNA per well. Empty pCMX vector was used as a no receptor control. The same transfection plan was followed for a replicate set of plates for downstream protein analysis by immunoblotting. Cells were lysed 48 h later and luciferase activity was measured in Glo-Max Microplate reader (Promega) using the Dual Luciferase assay reporter system (Promega). Luciferase data was obtained by normalizing Firefly activity to Renilla control activity and fold change induction was calculated relative to activity in eGFP control cells.

### Statistical analysis

A Student t-test was used for comparisons between two groups with normal data distribution (for real time qPCR, MTS, and xenograft assays). A nonparametric method (Wilcoxon Rank Sum test) was used when data were not normally distributed (for the liver tumor burden analysis). In the Wilcoxon Rank Sum test, the *Src-2*^*+/+*^ group served as the reference, and was compared to either the *Src-2*^*-/-*^ or *Src-2*^*+/-*^ groups (multiple comparisons were not adjusted). SAS 9.4 TS Level 1M2 (Cary, NC) was used for data analysis. For survival analysis (S12 Fig), survival functions were constructed using Kaplan-Meier method and were compared using the log-rank test.

## Supporting information

S1 Table Genes that overlap in RNA-seq and ChIP-Seq datasets. List of 47 downregulated genes in *Src2*^*-/-*^ liver tumors and directly bound by SRC-2 in mouse liver.
(PDF)

S2 Table Analysis of somatic mutations in SRC-2 target genes in human tumors. Analysis of mutations in human tumors was performed using the COSMIC database (v77 release) [69].
(PDF)

S3 Table Summary of alterations in SRC-2 targets in human liver cancer. Analysis of missense mutations, deletions, and gene expression alterations in *NR0B2/SHP*, *DKK4*, *THRSP*, and *CADM4* in multiple liver cancer datasets.
(PDF)

S4 Table Quantitative real-time PCR primer sequences. (PDF)

S1 Fig Generation of *Src2*^*-/-*^*; tet-o-MYC; LAPtTA* mice. (A) Breeding scheme designed to generate experimental mice. (B) Western blot depicting absence of SRC2 protein in *Src2*^*-/-*^*; tet-o-MYC; LAPtTA* mice. Tubulin was used as a loading control.
(TIF)

S2 Fig Deficiency in glycogen storage in the livers of *Src2*^*-/-*^*; tet-o-MYC; LAPtTA* animals. Periodic acid-Schiff (PAS) staining performed on normal liver and tumors from *Src2*^*+/+*^ and *Src2*^*-/-*^ mice. A positive purple staining was observed (black arrows) in *Src2*^*-/-*^*; tet-o-MYC; LAPtTA* liver tumors.
(TIF)

S3 Fig Expression analysis of direct SRC-2 targets in a panel of human HCCs. A gene expression profiling dataset (GSE1898) was analyzed using GEO2R to generate individual gene expression profiles for SRC-2 target genes across 91 human HCCs relative to a pooled normal liver reference. Student’s t-test was performed to assess statistical significance, ** = p<0.01; *** = p<0.001; **** = p<0.0001.
(TIF)

S4 Fig Analysis of *Shp*, *Dkk4*, *Cadm4*, and *Thrsp* in an independent set of *Src-2*^-/-^ and *Src-2*^+/+^ liver tumors. Real-time PCR quantification of *Shp*, *Dkk4*, *Cadm4*, and *Thrsp* expression in four independent liver tumors from *Src-2*^-/-^ and *Src-2*^+/+^ mice. Bar graphs represent mRNA expression normalized to *Actin* and error bars represent SDs from triplicate measurements (n = 4 tumors per group). Student’s t-test was performed to assess statistical significance (* = p<0.05).
(TIF)

S5 Fig *Vegfc*, *Fgf1 and Masp1* as putative SRC-2 target genes. Left, Real-time PCR quantification of *Vegfc*, *Fgf1 and Masp1* in *Src2*^*+/+*^ and *Src2*^-/-^ liver tumors. Bar graphs represent mRNA expression normalized to *ACTIN* and error bars represent SDs from triplicate measurements measured in five tumors per group. Student’s t-test was performed to assess statistical significance. Right, mouse liver SRC-2 ChIP-Seq peaks depicting SRC-2 binding sites in promoter regions of *Vegfc*, *Fgf1* and *Masp1*.
(TIF)

S6 Fig MYC expression is comparable in human liver cancer cells and *Src-2*^*-/-*^*; tet-o-MYC; LAPtTA* liver tumors. Western blot analysis depicting MYC protein levels in a panel of human liver cancer cells and a liver tumor from an *Src-2*^*-/-*^*; tet-o-MYC; LAPtTA* animal (after dox removal, with MYC overexpression). Of note, MYC levels were not experimentally modulated in any of the human liver cancer cell lines used in these studies.
(TIF)

S7 Fig Analysis of *Cyp7a1* in *Src-2*^*-/-*^ liver tumors and CYCLIN D1 in *SHP* shRNA cells and tumors. (A) Real-time PCR quantification of *Cyp7a1* expression in *Src-2*^*+/+*^ and *Src-2*^*-/-*^ liver tumors. Bar graphs represent mRNA expression of *Cyp7a1* normalized to *ACTIN* and error bars represent SDs from triplicate measurements (n = 4 tumors per group). Student’s t-test was performed to assess statistical significance (* = p<0.05). (B) Real-time PCR quantification of *CYCLIN D1* expression levels in *SHP* shRNA Huh7 cells. Bar graphs represent mRNA expression of *CYCLIN D1* normalized to *ACTIN* and error bars represent SDs from triplicate measurements. Student’s t-test was performed to assess statistical significance. (C) Western blot analysis depicting CYCLIN D1 protein levels in *SHP* shRNA and control shRNA cells. (D) Real-time PCR quantification of *CYCLIN D1* expression levels in tumors derived from xenograft assays with control or *SHP* shRNA-1. Bar graphs represent mRNA expression of *CYCLIN D1* normalized to *ACTIN* and error bars represent SDs from triplicate measurements (n = 4 tumors per group). Student’s t-test was performed to assess statistical significance (**** = p<0.0001).
(TIF)

S8 Fig SRC-2 inhibition reduces target gene expression and increases cell proliferation of Huh7 cells. (A) Real-time PCR quantification of *SRC-2*, *SHP*, and *DKK4* expression in Huh7 cells after inhibition of *SRC-2* with two independent shRNAs. Bar graphs represent mRNA expression of the labeled transcript normalized to *ACTIN* and error bars represent SDs from triplicate measurements. (B) MTS assay measuring proliferation of Huh7 cells with control shRNA, *SRC-2* shRNA-1, or *SRC-2* shRNA-2. Error bars in real-time quantitation and proliferation assays represent SDs from triplicate measurements. A student’s t-test was performed to determine statistical significance. * = p<0.05; ** = p<0.01; *** = p<0.001; **** = p<0.0001.
(TIF)

S9 Fig THRSP and DKK4 are not sufficient to rescue enhanced tumor burden upon SRC-2 inhibition. (A) Western blot analysis demonstrating inhibition of SRC-2 targets in HepG2 cells expressing SRC-2 shRNA-1, as compared to control shRNA cells, and overexpression of each of the four targets alone or in combination in *SRC-2* shRNA-1 cells. The V5 antibody detects V5-tagged SHP in the rescue experiment, but does not recognize endogenous SHP in HepG2 cells. (B) MTS assay measuring proliferation of HepG2 cells with control shRNA, *SRC-2* shRNA-1, or *SRC-2* shRNA-1 with overexpression of *THRSP* or *DKK4* alone. (C) Quantification of tumor volumes in nude mice injected with HepG2 cells as described in (B). Bars represent mean tumor volumes. Error bars in proliferation assays represent SDs from triplicate measurements. Error bars in xenograft experiments represent SDs from a total of ten subcutaneous injections (n = 5 mice) per shRNA tested. A student’s t-test was performed to determine statistical significance. * = p<0.05; ** = p<0.01; *** = p<0.001. Black asterisks represent comparisons to the control shRNA. Red asterisks represent comparisons to SRC-2 shRNA-1.
(TIF)

S10 Fig Putative nuclear receptor (NR) binding sites in the promoter regions of SRC-2 target genes. Depiction of NR binding motifs in the promoter regions of *Dkk4* (A),*Thrsp* (B), *Cadm4* (C), and *Shp* (D) as predicted by NHRscan. SRC-2 ChIP-Seq peaks for *Shp*, *Dkk4*, *Thrsp*, and *Cadm4* are depicted with SRC-2 binding sites represented by red bars as well as nucleotide consensus sequences corresponding to putative NR binding motifs.
(TIF)

S11 Fig SRC-2 cooperates with FXR to activate SHP reporter activity in liver cancer cells. (A) eGFP control or SRC-2 expressing Huh7 cells were transfected with FXR plasmid (20ng) in combination with *SHP*-LUC or *SHP*^Δ215-569^-LUC reporter plasmids (80ng), 1 ng Renilla control reporter plasmid and 199ng pUC19 plasmid yielding a total of 300 ng DNA per well, and then measured for luciferase activity after 48 hours. (B) Western blot demonstrating expression of FXR in Huh7 cells transfected with *SHP*-LUC plasmids with and without FXR. Numbers in red represent quantification of FXR protein levels relative to the *SHP*-LUC only sample and normalized to Tubulin. (C) Quantification of *SHP*-luciferase fold-induction in eGFP control and SRC-2 expressing cells.
(TIF)

S12 Fig CBioPortal analysis of *SRC-2 (NCOA2)* alterations and patient survival in HCC. (A) Oncoprints demonstrating mRNA upregulation, amplification, and missense mutations of *SRC-2* in 442 HCC tumors from the provisional TCGA dataset. (B) Kaplan-Meier survival analysis of HCC patients with missense mutations, mRNA upregulation, and amplification of *SRC-2*.
(TIF)

## References

[1] Sengupta B, Siddiqi SA. Hepatocellular carcinoma: important biomarkers and their significance in molecular diagnostics and therapy. Curr Med Chem. 2012;19(22):3722–9. 22680921 10.2174/092986712801661059 PMC11447867

[2] Flores A, Marrero JA. Emerging trends in hepatocellular carcinoma: focus on diagnosis and therapeutics. Clin Med Insights Oncol. 2014;8:71–6. 10.4137/CMO.S9926 24899827 PMC4039215

[3] Strumberg D. Preclinical and clinical development of the oral multikinase inhibitor sorafenib in cancer treatment. Drugs Today (Barc). 2005;41(12):773–84. 16474853 10.1358/dot.2005.41.12.937959

[4] Hansen LJ, Tennant BC, Seeger C, Ganem D. Differential activation of myc gene family members in hepatic carcinogenesis by closely related hepatitis B viruses. Mol Cell Biol. 1993;13(1):659–67. 8380230 10.1128/mcb.13.1.659-667.1993 PMC358944

[5] Ozen C, Yildiz G, Dagcan AT, Cevik D, Ors A, Keles U, et al Genetics and epigenetics of liver cancer. N Biotechnol. 2013;30(4):381–4. 10.1016/j.nbt.2013.01.007 23392071

[6] Totoki Y, Tatsuno K, Covington KR, Ueda H, Creighton CJ, Kato M, et al Trans-ancestry mutational landscape of hepatocellular carcinoma genomes. Nat Genet. 2014;46(12):1267–73. 10.1038/ng.3126 25362482

[7] Collier LS, Largaespada DA. Transposons for cancer gene discovery: Sleeping Beauty and beyond. Genome Biol. 2007;8 Suppl 1:S15. 18047692 10.1186/gb-2007-8-s1-s15 PMC2106843

[8] Copeland NG, Jenkins NA. Harnessing transposons for cancer gene discovery. Nat Rev Cancer. 2010;10(10):696–706. 10.1038/nrc2916 20844553

[9] Dupuy AJ. Transposon-based screens for cancer gene discovery in mouse models. Semin Cancer Biol. 2010;20(4):261–8. 10.1016/j.semcancer.2010.05.003 20478384 PMC2940989

[10] Dupuy AJ, Jenkins NA, Copeland NG. Sleeping beauty: a novel cancer gene discovery tool. Hum Mol Genet. 2006;15 Spec No 1:R75–9. 10.1093/hmg/ddl061 16651372

[11] Riordan JD, Keng VW, Tschida BR, Scheetz TE, Bell JB, Podetz-Pedersen KM, et al Identification of rtl1, a retrotransposon-derived imprinted gene, as a novel driver of hepatocarcinogenesis. PLoS Genet. 2013;9(4):e1003441 10.1371/journal.pgen.1003441 23593033 PMC3616914

[12] Tschida BR, Largaespada DA, Keng VW. Mouse models of cancer: Sleeping Beauty transposons for insertional mutagenesis screens and reverse genetic studies. Semin Cell Dev Biol. 2014;27:86–95. 10.1016/j.semcdb.2014.01.006 24468652 PMC4035448

[13] O'Donnell KA, Keng VW, York B, Reineke EL, Seo D, Fan D, et al A Sleeping Beauty mutagenesis screen reveals a tumor suppressor role for Ncoa2/Src-2 in liver cancer. Proc Natl Acad Sci U S A. 2012;109(21):E1377–86. 10.1073/pnas.1115433109 22556267 PMC3361419

[14] Chopra AR, Kommagani R, Saha P, Louet JF, Salazar C, Song J, et al Cellular energy depletion resets whole-body energy by promoting coactivator-mediated dietary fuel absorption. Cell Metab. 2011;13(1):35–43. 10.1016/j.cmet.2010.12.001 21195347 PMC3072049

[15] Chopra AR, Louet JF, Saha P, An J, Demayo F, Xu J, et al Absence of the SRC-2 coactivator results in a glycogenopathy resembling Von Gierke's disease. Science. 2008;322(5906):1395–9. 10.1126/science.1164847 19039140 PMC2668604

[16] Jeong JW, Kwak I, Lee KY, White LD, Wang XP, Brunicardi FC, et al The genomic analysis of the impact of steroid receptor coactivators ablation on hepatic metabolism. Mol Endocrinol. 2006;20(5):1138–52. 10.1210/me.2005-0407 16423883

[17] Stashi E, Lanz RB, Mao J, Michailidis G, Zhu B, Kettner NM, et al SRC-2 is an essential coactivator for orchestrating metabolism and circadian rhythm. Cell Rep. 2014;6(4):633–45. 10.1016/j.celrep.2014.01.027 24529706 PMC4096300

[18] Xu J, Li Q. Review of the in vivo functions of the p160 steroid receptor coactivator family. Mol Endocrinol. 2003;17(9):1681–92. 10.1210/me.2003-0116 12805412

[19] Ye X, Han SJ, Tsai SY, DeMayo FJ, Xu J, Tsai MJ, et al Roles of steroid receptor coactivator (SRC)-1 and transcriptional intermediary factor (TIF) 2 in androgen receptor activity in mice. Proc Natl Acad Sci U S A. 2005;102(27):9487–92. 10.1073/pnas.0503577102 15983373 PMC1172261

[20] York B, O'Malley BW. Steroid receptor coactivator (SRC) family: masters of systems biology. J Biol Chem. 2010;285(50):38743–50. 10.1074/jbc.R110.193367 20956538 PMC2998129

[21] Fleet T, Zhang B, Lin F, Zhu B, Dasgupta S, Stashi E, et al SRC-2 orchestrates polygenic inputs for fine-tuning glucose homeostasis. Proc Natl Acad Sci U S A. 2015;112(44):E6068–77. 10.1073/pnas.1519073112 26487680 PMC4640775

[22] Zhu B, Gates LA, Stashi E, Dasgupta S, Gonzales N, Dean A, et al Coactivator-Dependent Oscillation of Chromatin Accessibility Dictates Circadian Gene Amplitude via REV-ERB Loading. Mol Cell. 2015;60(5):769–83. 10.1016/j.molcel.2015.10.024 26611104 PMC4671835

[23] Lee PJ. Glycogen storage disease type I: pathophysiology of liver adenomas. Eur J Pediatr. 2002;161 Suppl 1:S46–9. 12373570 10.1007/s00431-002-1002-0

[24] Fenne IS, Helland T, Flageng MH, Dankel SN, Mellgren G, Sagen JV. Downregulation of steroid receptor coactivator-2 modulates estrogen-responsive genes and stimulates proliferation of mcf-7 breast cancer cells. PLoS One. 2013;8(7):e70096 10.1371/journal.pone.0070096 23936147 PMC3728357

[25] Ahn SM, Jang SJ, Shim JH, Kim D, Hong SM, Sung CO, et al Genomic portrait of resectable hepatocellular carcinomas: implications of RB1 and FGF19 aberrations for patient stratification. Hepatology. 2014;60(6):1972–82. 10.1002/hep.27198 24798001

[26] Gao J, Aksoy BA, Dogrusoz U, Dresdner G, Gross B, Sumer SO, et al Integrative analysis of complex cancer genomics and clinical profiles using the cBioPortal. Sci Signal. 2013;6(269):pl1. 10.1126/scisignal.2004088 PMC4160307 23550210

[27] Dasgupta S, Putluri N, Long W, Zhang B, Wang J, Kaushik AK, et al Coactivator SRC-2-dependent metabolic reprogramming mediates prostate cancer survival and metastasis. J Clin Invest. 2015;125(3):1174–88. 10.1172/JCI76029 25664849 PMC4362260

[28] Shachaf CM, Kopelman AM, Arvanitis C, Karlsson A, Beer S, Mandl S, et al MYC inactivation uncovers pluripotent differentiation and tumour dormancy in hepatocellular cancer. Nature. 2004;431(7012):1112–7. 10.1038/nature03043 15475948

[29] Mukherjee A, Soyal SM, Fernandez-Valdivia R, Gehin M, Chambon P, Demayo FJ, et al Steroid receptor coactivator 2 is critical for progesterone-dependent uterine function and mammary morphogenesis in the mouse. Mol Cell Biol. 2006;26(17):6571–83. 10.1128/MCB.00654-06 16914740 PMC1592830

[30] Lee JS, Heo J, Libbrecht L, Chu IS, Kaposi-Novak P, Calvisi DF, et al A novel prognostic subtype of human hepatocellular carcinoma derived from hepatic progenitor cells. Nat Med. 2006;12(4):410–6. 10.1038/nm1377 16532004

[31] Sandhu DS, Baichoo E, Roberts LR. Fibroblast growth factor signaling in liver carcinogenesis. Hepatology. 2014;59(3):1166–73. 24716202 10.1002/hep.26679

[32] Tacconi C, Correale C, Gandelli A, Spinelli A, Dejana E, D'Alessio S, et al Vascular endothelial growth factor C disrupts the endothelial lymphatic barrier to promote colorectal cancer invasion. Gastroenterology. 2015;148(7):1438–51 e8. 10.1053/j.gastro.2015.03.005 25754161

[33] Rutkowski MJ, Sughrue ME, Kane AJ, Mills SA, Parsa AT. Cancer and the complement cascade. Mol Cancer Res. 2010;8(11):1453–65. 10.1158/1541-7786.MCR-10-0225 20870736

[34] Takahashi M, Iwaki D, Kanno K, Ishida Y, Xiong J, Matsushita M, et al Mannose-binding lectin (MBL)-associated serine protease (MASP)-1 contributes to activation of the lectin complement pathway. J Immunol. 2008;180(9):6132–8. 18424734 10.4049/jimmunol.180.9.6132

[35] Cvoro A, Tatomer D, Tee MK, Zogovic T, Harris HA, Leitman DC. Selective estrogen receptor-beta agonists repress transcription of proinflammatory genes. J Immunol. 2008;180(1):630–6. 18097065 10.4049/jimmunol.180.1.630

[36] Sun Y, Tao YG, Kagan BL, He Y, S S Jr. Modulation of transcription parameters in glucocorticoid receptor-mediated repression. Mol Cell Endocrinol. 2008;295(1–2):59–69. 10.1016/j.mce.2008.05.008 18583028 PMC2662735

[37] Seol W, Choi HS, Moore DD. An orphan nuclear hormone receptor that lacks a DNA binding domain and heterodimerizes with other receptors. Science. 1996;272(5266):1336–9. 8650544 10.1126/science.272.5266.1336

[38] Lee HK, Lee YK, Park SH, Kim YS, Park SH, Lee JW, et al Structure and expression of the orphan nuclear receptor SHP gene. J Biol Chem. 1998;273(23):14398–402. 9603951 10.1074/jbc.273.23.14398

[39] Kerr TA, Saeki S, Schneider M, Schaefer K, Berdy S, Redder T, et al Loss of nuclear receptor SHP impairs but does not eliminate negative feedback regulation of bile acid synthesis. Dev Cell. 2002;2(6):713–20. 12062084 10.1016/s1534-5807(02)00154-5 PMC4010195

[40] Zhang Y, Xu P, Park K, Choi Y, Moore DD, Wang L. Orphan receptor small heterodimer partner suppresses tumorigenesis by modulating cyclin D1 expression and cellular proliferation. Hepatology. 2008;48(1):289–98. 10.1002/hep.22342 18537191 PMC3800167

[41] Li G, Kong B, Zhu Y, Zhan L, Williams JA, Tawfik O, et al Small heterodimer partner overexpression partially protects against liver tumor development in farnesoid X receptor knockout mice. Toxicol Appl Pharmacol. 2013;272(2):299–305. 10.1016/j.taap.2013.06.016 23811326 PMC4039201

[42] Fatima S, Lee NP, Tsang FH, Kolligs FT, Ng IO, Poon RT, et al Dickkopf 4 (DKK4) acts on Wnt/beta-catenin pathway by influencing beta-catenin in hepatocellular carcinoma. Oncogene. 2012;31(38):4233–44. 10.1038/onc.2011.580 22249261

[43] Fukuhara H, Kuramochi M, Nobukuni T, Fukami T, Saino M, Maruyama T, et al Isolation of the TSLL1 and TSLL2 genes, members of the tumor suppressor TSLC1 gene family encoding transmembrane proteins. Oncogene. 2001;20(38):5401–7. 10.1038/sj.onc.1204696 11536053

[44] LaFave LT, Augustin LB, Mariash CN. S14: insights from knockout mice. Endocrinology. 2006;147(9):4044–7. 10.1210/en.2006-0473 16809440

[45] Sandelin A, Wasserman WW. Prediction of nuclear hormone receptor response elements. Mol Endocrinol. 2005;19(3):595–606. 10.1210/me.2004-0101 15563547

[46] Paquette MA, Atlas E, Wade MG, Yauk CL. Thyroid hormone response element half-site organization and its effect on thyroid hormone mediated transcription. PLoS One. 2014;9(6):e101155 10.1371/journal.pone.0101155 24971931 PMC4074170

[47] Grontved L, Waterfall JJ, Kim DW, Baek S, Sung MH, Zhao L, et al Transcriptional activation by the thyroid hormone receptor through ligand-dependent receptor recruitment and chromatin remodelling. Nat Commun. 2015;6:7048 10.1038/ncomms8048 25916672 PMC6309829

[48] Liao CH, Yeh CT, Huang YH, Wu SM, Chi HC, Tsai MM, et al Dickkopf 4 positively regulated by the thyroid hormone receptor suppresses cell invasion in human hepatoma cells. Hepatology. 2012;55(3):910–20. 10.1002/hep.24740 21994129

[49] Rastinejad F, Wagner T, Zhao Q, Khorasanizadeh S. Structure of the RXR-RAR DNA-binding complex on the retinoic acid response element DR1. EMBO J. 2000;19(5):1045–54. 10.1093/emboj/19.5.1045 10698945 PMC305643

[50] Chambon P. A decade of molecular biology of retinoic acid receptors. FASEB J. 1996;10(9):940–54. 8801176

[51] Thomas AM, Hart SN, Kong B, Fang J, Zhong XB, Guo GL. Genome-wide tissue-specific farnesoid X receptor binding in mouse liver and intestine. Hepatology. 2010;51(4):1410–9. 10.1002/hep.23450 20091679 PMC4855519

[52] Lu TT, Makishima M, Repa JJ, Schoonjans K, Kerr TA, Auwerx J, et al Molecular basis for feedback regulation of bile acid synthesis by nuclear receptors. Mol Cell. 2000;6(3):507–15. 11030331 10.1016/s1097-2765(00)00050-2

[53] Aragane H, Sakakura C, Nakanishi M, Yasuoka R, Fujita Y, Taniguchi H, et al Chromosomal aberrations in colorectal cancers and liver metastases analyzed by comparative genomic hybridization. Int J Cancer. 2001;94(5):623–9. 11745455 10.1002/ijc.1522

[54] Chiang DY, Villanueva A, Hoshida Y, Peix J, Newell P, Minguez B, et al Focal gains of VEGFA and molecular classification of hepatocellular carcinoma. Cancer Res. 2008;68(16):6779–88. 10.1158/0008-5472.CAN-08-0742 18701503 PMC2587454

[55] Parada LA, Hallen M, Tranberg KG, Hagerstrand I, Bondeson L, Mitelman F, et al Frequent rearrangements of chromosomes 1, 7, and 8 in primary liver cancer. Genes Chromosomes Cancer. 1998;23(1):26–35. 9713994 10.1002/(sici)1098-2264(199809)23:1<26::aid-gcc5>3.3.co;2-g

[56] Lee JS, Chu IS, Mikaelyan A, Calvisi DF, Heo J, Reddy JK, et al Application of comparative functional genomics to identify best-fit mouse models to study human cancer. Nat Genet. 2004;36(12):1306–11. 10.1038/ng1481 15565109

[57] Park YY, Choi HS, Lee JS. Systems-level analysis of gene expression data revealed NR0B2/SHP as potential tumor suppressor in human liver cancer. Mol Cells. 2010;30(5):485–91. 10.1007/s10059-010-0136-6 20853064

[58] Jeong Y, Xie Y, Xiao G, Behrens C, Girard L, Wistuba II, et al Nuclear receptor expression defines a set of prognostic biomarkers for lung cancer. PLoS Med. 2010;7(12):e1000378 10.1371/journal.pmed.1000378 21179495 PMC3001894

[59] Chi HC, Liao CH, Huang YH, Wu SM, Tsai CY, Liao CJ, et al Thyroid hormone receptor inhibits hepatoma cell migration through transcriptional activation of Dickkopf 4. Biochem Biophys Res Commun. 2013;439(1):60–5. 10.1016/j.bbrc.2013.08.028 23958302

[60] Raveh S, Gavert N, Spiegel I, Ben-Ze'ev A. The cell adhesion nectin-like molecules (Necl) 1 and 4 suppress the growth and tumorigenic ability of colon cancer cells. J Cell Biochem. 2009;108(1):326–36. 10.1002/jcb.22258 19565570

[61] Williams YN, Masuda M, Sakurai-Yageta M, Maruyama T, Shibuya M, Murakami Y. Cell adhesion and prostate tumor-suppressor activity of TSLL2/IGSF4C, an immunoglobulin superfamily molecule homologous to TSLC1/IGSF4. Oncogene. 2006;25(10):1446–53. 10.1038/sj.onc.1209192 16261159

[62] Jump DB, Oppenheimer JH. High basal expression and 3,5,3'-triiodothyronine regulation of messenger ribonucleic acid S14 in lipogenic tissues. Endocrinology. 1985;117(6):2259–66. 10.1210/endo-117-6-2259 4065033

[63] Sanchez-Rodriguez J, Kaninda-Tshilumbu JP, Santos A, Perez-Castillo A. The spot 14 protein inhibits growth and induces differentiation and cell death of human MCF-7 breast cancer cells. Biochem J. 2005;390(Pt 1):57–65. 10.1042/BJ20042080 15819613 PMC1188266

[64] Vella KR, Ramadoss P, Costa ESRH, Astapova I, Ye FD, Holtz KA, et al Thyroid hormone signaling in vivo requires a balance between coactivators and corepressors. Mol Cell Biol. 2014;34(9):1564–75. 10.1128/MCB.00129-14 24550004 PMC3993596

[65] Stashi E, York B, O'Malley BW. Steroid receptor coactivators: servants and masters for control of systems metabolism. Trends Endocrinol Metab. 2014;25(7):337–47. 10.1016/j.tem.2014.05.004 24953190 PMC4108168

[66] Wang L, Yu Y, Chow DC, Yan F, Hsu CC, Stossi F, et al Characterization of a Steroid Receptor Coactivator Small Molecule Stimulator that Overstimulates Cancer Cells and Leads to Cell Stress and Death. Cancer Cell. 2015;28(2):240–52. 10.1016/j.ccell.2015.07.005 26267537 PMC4536575

[67] Meyer CA, Liu XS. Identifying and mitigating bias in next-generation sequencing methods for chromatin biology. Nat Rev Genet. 2014;15(11):709–21. 10.1038/nrg3788 25223782 PMC4473780

[68] McLean CY, Bristor D, Hiller M, Clarke SL, Schaar BT, Lowe CB, et al GREAT improves functional interpretation of cis-regulatory regions. Nat Biotechnol. 2010;28(5):495–501. 10.1038/nbt.1630 20436461 PMC4840234

[69] Forbes SA, Beare D, Gunasekaran P, Leung K, Bindal N, Boutselakis H, et al COSMIC: exploring the world's knowledge of somatic mutations in human cancer. Nucleic Acids Res. 2015;43(Database issue):D805–11. 10.1093/nar/gku1075 25355519 PMC4383913

