# Correction: SRC-2-mediated coactivation of anti-tumorigenic target genes suppresses MYC-induced liver cancer

**DOI:** 10.1371/journal.pgen.1007344

**Published:** 2018-04-11

**Authors:** Shruthy Suresh, Deniz Durakoglugil, Xiaorong Zhou, Bokai Zhu, Sarah A. Comerford, Chao Xing, Xian-Jin Xie, Brian York, Kathryn A. O’Donnell

The following information is missing from the Funding section: This work was supported by The Cancer Prevention Research Institute of Texas (http://www.cprit.state.tx.us/) (RP160157 to SS).

## References

[1] Suresh S, Durakoglugil D, Zhou X, Zhu B, Comerford SA, Xing C, et al (2017) SRC-2-mediated coactivation of anti-tumorigenic target genes suppresses MYC-induced liver cancer. PLoS Genet 13(3): e1006650 https://doi.org/10.1371/journal.pgen.1006650 28273073 10.1371/journal.pgen.1006650 PMC5362238

