# Supplementary material for: SRC-2-mediated coactivation of anti-tumorigenic target genes suppresses MYC-induced liver cancer
Source: PLoS Genet. 2017 Mar 8;13(3):e1006650. doi: 10.1371/journal.pgen.1006650 (PMC5362238; doi:10.1371/journal.pgen.1006650)
Supplement: S1 Table — List of 47 downregulated genes in Src2-/- liver tumors and directly bound by SRC-2 in mouse liver. (PDF) [file pgen.1006650.s001.pdf]

S1 Table. Genes that overlap in RNA-Seq and ChIP-Seq datasets

List of 47 downregulated genes in SRC-2 KO liver tumors and directly bound by SRC-2 in mouse liver

MOUSE GENE ID	HUMAN GENE ID	Fold Decrease	p-value	q-value
Mug2		7.03	5.00E-05	0.0076
Slc22a7	*SLC22A7*	5.7316	2.00E-04	0.02396
Cyp21a1	*CYP21AIP*	4.2942	5.00E-05	0.0076
Cyp2c29	*CYP2C8*	4.004	5.00E-05	0.0076
Scd1	*SCD1*	3.9866	5.00E-05	0.0076
Chrna4	*CHRNA4*	3.636	0.00035	0.03585
Nr0b2	*SHP*	3.497	0.01515	0.43285
Dio1	*DIO1*	3.35	0.00085	0.06788
Cxcl1	*CXCL1*	3.281	5.00E-05	0.0076
Ncoa2	*NCOA2*	3.1	5.00E-05	0.0076
Cadm4	*CADM4*	2.9311	0.00025	0.02718
Cyp1a1	*CYP1A1*	2.8489	0.00165	0.1032
Thrsp	*THRSP*	2.805	5.00E-05	0.0076
Ccr7	*CCR7*	2.798	0.0034	0.15966
Rab25	*RAB25*	2.785	0.0173	0.46139
Cxcl2	*CXCL2*	2.7403	0.01075	0.34931
Ovol1	*OVOL1*	2.7229	0.00255	0.13399
Ier3	*IER3*	2.4307	0.00355	0.16561
Scml4	*SCML4*	2.4166	0.0052	0.21367
Cxcl10	*CXCL10*	2.3654	5.00E-05	0.0076
Abcg8	*SBCG8*	2.302	0.0019	0.11237
Igsf11	*IGSF11*	2.1678	5.00E-05	0.0076
Acacb	*ACACB*	2.16081	5.00E-05	0.0076
Glt1d1	*GLT1D1*	2.1524	0.00025	0.02718
Aloxe3	*ALOXE3*	2.0871	0.02665	0.59888
Dkk4	*DKK4*	2.0432	0.0357	0.69126
Socs3	*SOCS3*	2.0223	0.00065	0.05671
Serpine1	*SERPINE1*	2.019	5.00E-05	0.0076
Il33	*IL33*	1.9771	0.0388	0.71879
Mapkapk3	*MAPKAPK3*	1.9757	5.00E-05	0.0076

MOUSE GENE ID	HUMAN GENE ID	Fold Decrease	p-value	q-value
Gata6	*GATA6*	1.897	0.03425	0.67742
Tmem79	*TMEM79*	1.879	0.0178	0.47029
Cnksr1	*CNKSR1*	1.85233	0.00275	0.14084
Tm6sf2	*TM6SF2*	1.7702	0.0172	0.46139
Igfbp1	*IGFBP1*	1.7481	5.00E-05	0.0076
Egr1	*EGR1*	1.74384	0.0013	0.09052
Mospd3	*MOSPD3*	1.6902	0.0049	0.20675
Cldn6	*CLDN6*	1.6712	5.00E-05	0.0076
Mug1		1.5769	8.00E-04	0.06536
Mal2	*MAL2*	1.5614	0.00085	0.06788
Cblc	*CBLC*	1.5545	0.02895	0.61668
Ccbl2	*CCBL2*	1.549	0.00135	0.09174
Pmm1	*PMM1*	1.517	0.00135	0.09174
Hgfac	*HGFAC*	1.4755	0.0061	0.2343
Tat	*TAT*	1.458	0.02205	0.53923
Pemt	*PEMT*	1.4371	0.0208	0.51853
Acss2	*ACSS2*	1.4343	0.0222	0.54132
