# Supplementary material for: SRC-2-mediated coactivation of anti-tumorigenic target genes suppresses MYC-induced liver cancer
Source: PLoS Genet. 2017 Mar 8;13(3):e1006650. doi: 10.1371/journal.pgen.1006650 (PMC5362238; doi:10.1371/journal.pgen.1006650)
Supplement: S2 Table — Analysis of mutations in human tumors was performed using the COSMIC database (v77 release) [69]. (PDF) [file pgen.1006650.s002.pdf]

S2 Table. Analysis of somatic mutations in SRC-2 target genes in human tumors.

Gene	Chromosome	Mutations	Nonsense mutations	Missense mutations
NR0B2	1	31 (4 in Breast, 5 in Stomach, 4 in Uterine, 1 in Colon, 2 in GBM, 2 Head and Neck Cancers, 2 in Liver, 4 in Lung and 7 in Melanoma	1	30
DKK4	8	47 (15 in Colon, 7 in Melanoma, 6 in Uterine, 4 in Breast, 4 in Stomach, 3 in Lung, 3 in Bladder, 2 in Esophagus, 2 in Thyroid, 1 in Pancreas)	2	45
THRSP	11	21 (2 in Bladder, 2 in Breast, 2 in GBM, 5 in Melanoma, 2 in liver and 2 in Stomach	0	21
CADM4	19	43 (10 in Melanoma, 6 in Stomach, 6 in Lung, 4 in Uterine, 3 in Colon and 3 in Prostate)	7	36

*COSMIC database analysis (v77 release)
