# Supplementary material for: SRC-2-mediated coactivation of anti-tumorigenic target genes suppresses MYC-induced liver cancer
Source: PLoS Genet. 2017 Mar 8;13(3):e1006650. doi: 10.1371/journal.pgen.1006650 (PMC5362238; doi:10.1371/journal.pgen.1006650)
Supplement: S3 Table — Analysis of missense mutations, deletions, and gene expression alterations in NR0B2/SHP, DKK4, THRSP, and CADM4 in multiple liver cancer datasets. (PDF) [file pgen.1006650.s003.pdf]

S3 Table. Summary of alterations in SRC-2 targets in human liver cancer.

Gene	Missense mutations (Y/N)	Deletions	mRNA Downregulation	
	HCC TCGA Provisional*		GSE1898** Log2 (T/N)	Other dataset(s) with downregulation
NR0B2	Y, 0.9%	1.1%	-1.056	Chen *et al*, *MBoC 2002*
DKK4	N	3.4%	-0.046	Fatima *et al*, *Oncogene,* 2012
THRSP	Y, 0.7%	-	-2.700	
CADM4	Y, 0.2%	-	0.219	

*CBioPortal
**NCBI GEO, Lee JS et al, *Nat. Med 2006;* T: Tumor, N: Normal Liver
%: Percentage of cases with alteration
