# Supplementary material for: SRC-2-mediated coactivation of anti-tumorigenic target genes suppresses MYC-induced liver cancer
Source: PLoS Genet. 2017 Mar 8;13(3):e1006650. doi: 10.1371/journal.pgen.1006650 (PMC5362238; doi:10.1371/journal.pgen.1006650)
Supplement: S4 Table — (PDF) [file pgen.1006650.s004.pdf]

S4 Table. Quantitative real-time PCR primer sequences

Gene	Sequence	Exon-exon junction
Thbs1	5'-GCATCTTCACCAGGGATCTG-3'	3-4 Forward
Thbs1	5'- CTGGGGTGGTTCCAAAGAC -3'	3-4 Reverse
Thbs4	5'- TCCTCAAGTGACAACAGCAAA -3'	2-3 Forward
Thbs4	5'- GTTGAACACCACCAGATGGA -3'	2-3 Reverse
Cadm4	5'- ACGATGGGTCCATAGTCGTC -3'	2-3 Forward
Cadm4	5'- GATCTGATGGTGGGTGTCCT -3'	2-3 Reverse
Cldn7	5'- CTGTGGGGGAGATGACAAAG -3'	2-3 Forward
Cldn7	5'- TGACAATCTGATGACCAATCC -3'	2-3 Reverse
Cldn4	5'- ATGGTCATCAGCATCATCGT -3'	2 Forward
Cldn4	5'- CATGATCTTGGCCTTGACG -3'	2 Reverse
Gadd45b	5'- CCTCACCGTGGGGGTGTA -3'	2-3 Forward
Gadd45b	5'- TCTGCAGAGCGATATCATCC-3'	2-3 Reverse
Gramd4	5'- GAGACGAGATCCCCCTGAA -3'	2-3 Forward
Gramd4	5'- GTCCTGTTGAAGTCCTGCAC -3'	2-3 Reverse
Unc5b	5'- GGAGCTCTTCGGGAACTACC -3'	3-4 Forward
Unc5b	5'- GCAGAAGGACCTCATGATCC -3'	3-4 Reverse
Eda2r	5'- CCAAGAATGCATCCCATGTA -3'	5-6 Forward
Eda2r	5'- AGTGCAACAAGTGTGGCTTC -3'	5-6 Reverse
G6pc	5'- GCGCAGCAGGTGTATACTATG -3'	3-4 Forward
G6pc	5'- GCTGCACAGCCCAGAATC -3'	3-4 Reverse
Pck1	5'- GACTTTGAGAAAGCATTCAACG -3'	3-4 Forward
Pck1	5'- GCGAGTCTGTCAGTTCAATACC -3'	3-4 Reverse

Scd1	5'- AAAGAGAAGGGCGGAAAACT -3'	4-5 Forward
Scd1	5'- GTGGGCAGGATGAAGCAC -3'	4-5 Reverse
Acacb	5'- CCGTAATGAACGTGCCATC -3'	4-5 Forward
Acacb	5'- TATTGGGTCCTCCTGGGACA -3'	4-5 Reverse
Masp1	5'- GATTTCTCCAATGAGGAACGA -3'	3-4 Forward
Masp1	5'- GGTCACAGGACAGCTCTTCA -3'	3-4 Reverse
Cd24a	5'- CTAGGGCTGGGGTTGCTG -3'	1-2 Forward
Cd24a	5'- GGATTTGGGGAAGCAGAAAT -3'	1-2 Reverse
Ccl24	5'- AATTCCAGAAAACCGAGTGG -3'	2-3 Forward
Ccl24	5'- AGCAGCTTGGGGTCAGTACA -3'	2-3 Reverse
C1rb	5'- CTTCTGTTCCTGCCATCCTG -3'	4-5 Forward
C1rb	5'- GGCTGGAGACATAGCCTGAG -3'	4-5 Reverse
Bmp2	5'- AAGTCAGTGGGAGAGCTTCG -3'	3- Forward
Bmp2	5'- GAGACACCTGGGTTCTCCTCT -3'	3- Reverse
Vegfc	5'- CTACAGATGTGGGGGTTGCT -3'	3-4 Forward
Vegfc	5'- GACTGGTTTGGGGCCTTG -3'	3-4 Reverse
Fgf1	5'- GGGGCCACTTCTTGAGGAT -3'	2-3 Forward
Fgf1	5'- CCGGTCTCCGTACCCTTTAT -3'	2-3 Reverse
Gdf10	5'- CTCTCCCAAATCCTTTGACG -3'	2-3 Forward
Gdf10	5'- CACAGCTCTGACGATGCTCT -3'	2-3 Reverse
Shp	5'- AGCTGGGTCCCAAGGAGTAT -3'	1-2 Forward
Shp	5'- AGCCTCCTGTTGCAGGTGT -3'	1-2 Reverse
Dkk4	5'- GCCAGCAGAGGAAAACAGAC -3'	3-4 Forward
Dkk4	5'- AGGGCCACAGTCAGAGGTT -3'	3-4 Reverse

Thrsp	5'- ACGGAGCCCCTGATCTCTAT-3'	1-2 Forward
Thrsp	5'- TTTCGTTGCCAGCCACCT-3'	1-2 Reverse
NR0B2	5'- GCTTAGCCCCAAGGAATATG-3'	1-2 Forward
NR0B2	5'- CCAGTGAGCCTCCTGCTG-3'	1-2 Reverse
DKK4	5'- AGGAGGTGCCAGCGAGAT-3'	1-2 Forward
DKK4	5'- TTGCTCATCAAGCTGCCTTT-3'	1-2 Reverse
THRSP	5'- AAGGCCATCTGTGTGGATGT-3'	1- Forward
THRSP	5'- GCACTCTCGTCCTCGACTTC-3'	1- Reverse
CADM4	5'- CGAGGCGTCCAATAAGCAC-3'	2-3 Forward
CADM4	5'- CACAATGGCATAGGGAACC-3'	2-3 Reverse
SRC2	5'- CGCAGCATGAAGGAGAATC -3'	9-10 Forward
SRC2	5'- GCAACAAGAGTGCCATCAGA -3'	9-10 Reverse
CCND1	5'- CCTGTCCTACTACCGCCTCA-3'	4-5 Forward
CCND1	5'- TGACTCCAGCAGGGCTTC-3'	4-5 Reverse
mActin	5'-CGGTTCCGATGCCCTGAGGCTCTT-3'	4-5 Forward
mActin	5'-CGTCACACTTCATGATGGAATTGA-3'	4-5 Reverse
hACTIN	5'-ATTGCCGACAGGATGCAGAA-3'	5-6 Forward
hACTIN	5'-ACATCTGCTGGAAGGTGGACAG -3'	5-6 Reverse
