# Supplementary figures and images for: SRC-2-mediated coactivation of anti-tumorigenic target genes suppresses MYC-induced liver cancer
Source: PLoS Genet. 2017 Mar 8;13(3):e1006650. doi: 10.1371/journal.pgen.1006650 (PMC5362238; doi:10.1371/journal.pgen.1006650)

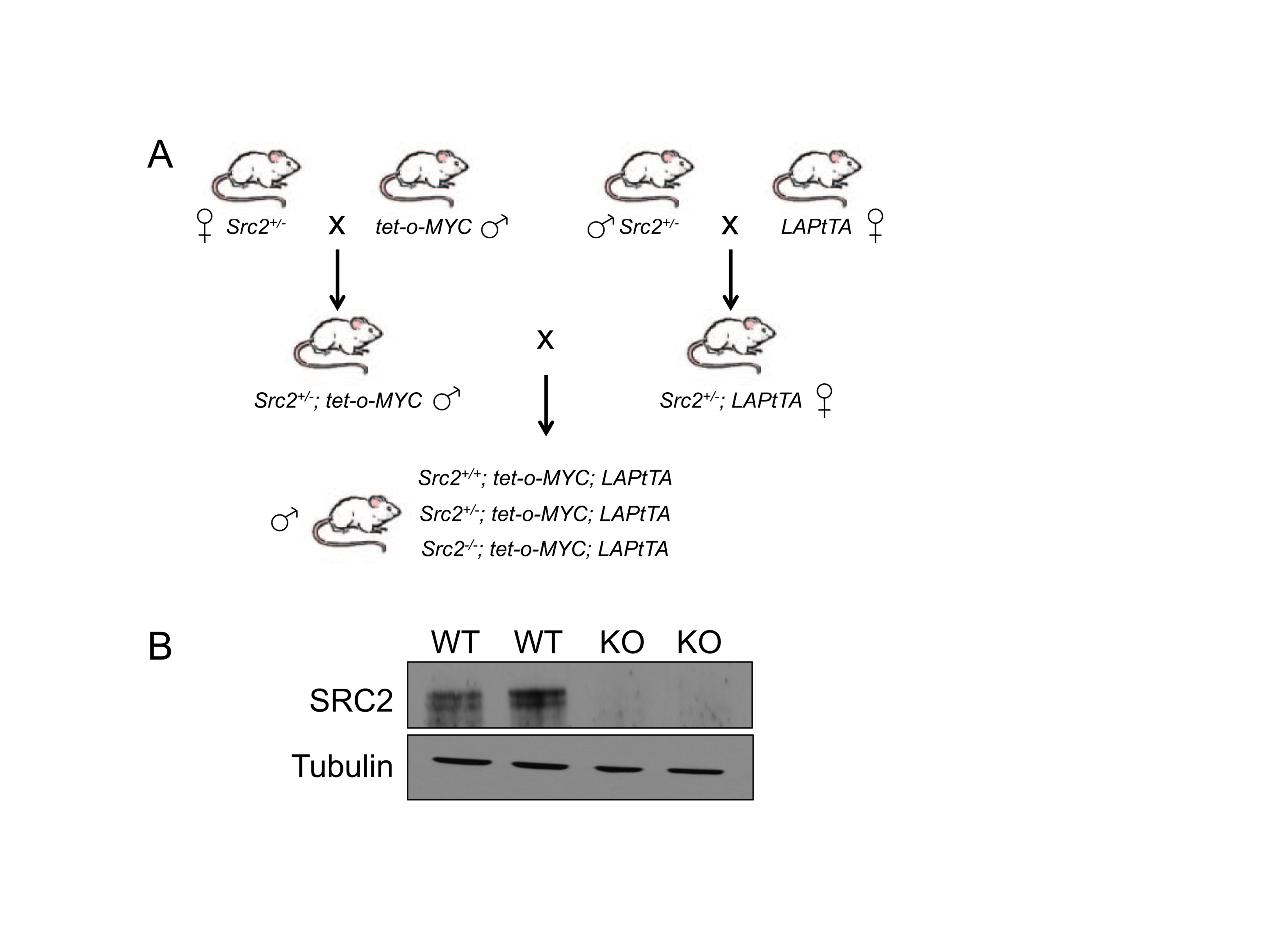

Supplement: S1 Fig — (A) Breeding scheme designed to generate experimental mice. (B) Western blot depicting absence of SRC2 protein in Src2-/-; tet-o-MYC; LAPtTA mice. Tubulin was used as a loading control. (TIF) [file pgen.1006650.s005.tif]

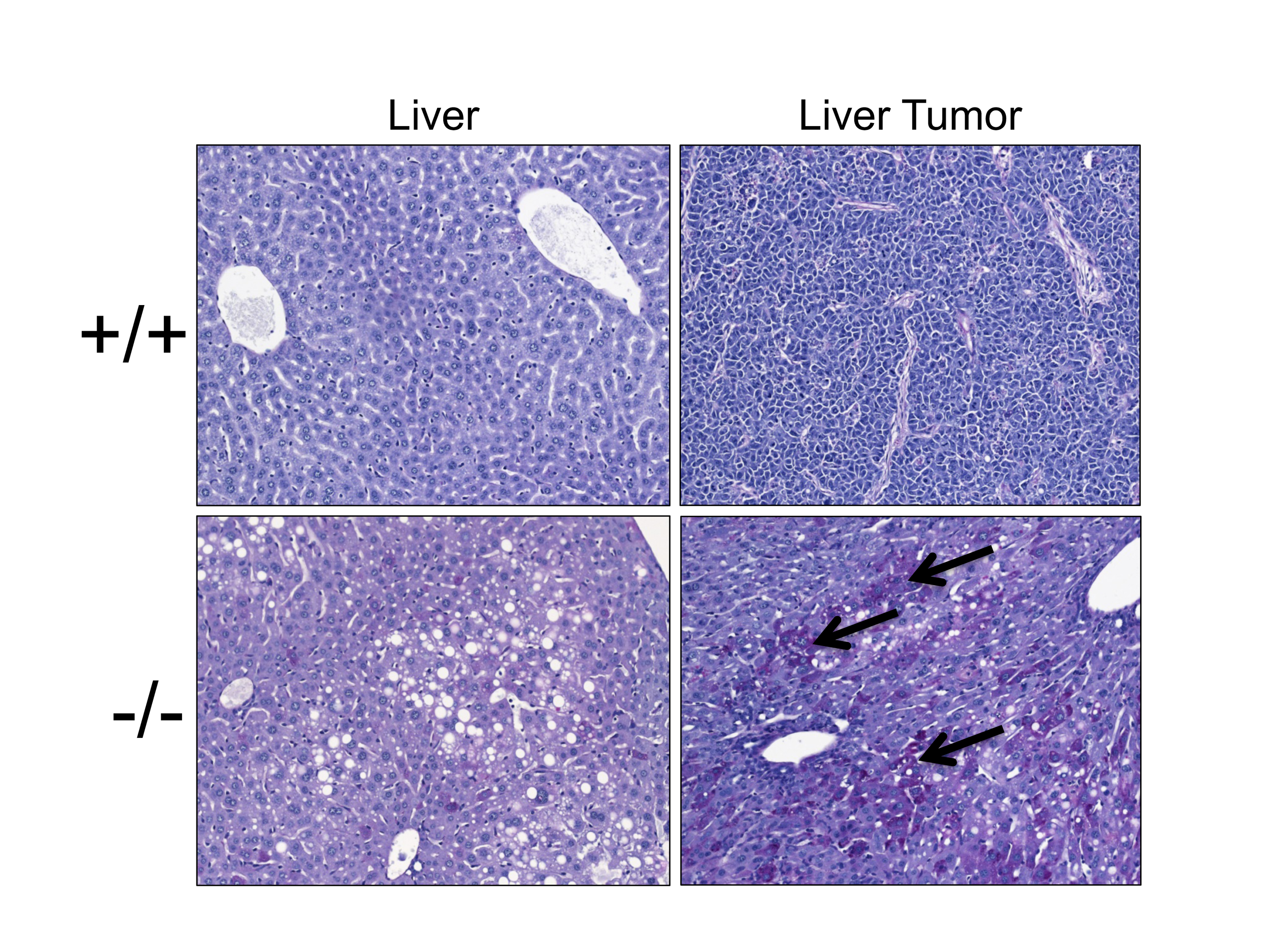

Supplement: S2 Fig — Periodic acid-Schiff (PAS) staining performed on normal liver and tumors from Src2+/+ and Src2-/- mice. A positive purple staining was observed (black arrows) in Src2-/-; tet-o-MYC; LAPtTA liver tumors. (TIF) [file pgen.1006650.s006.tif]

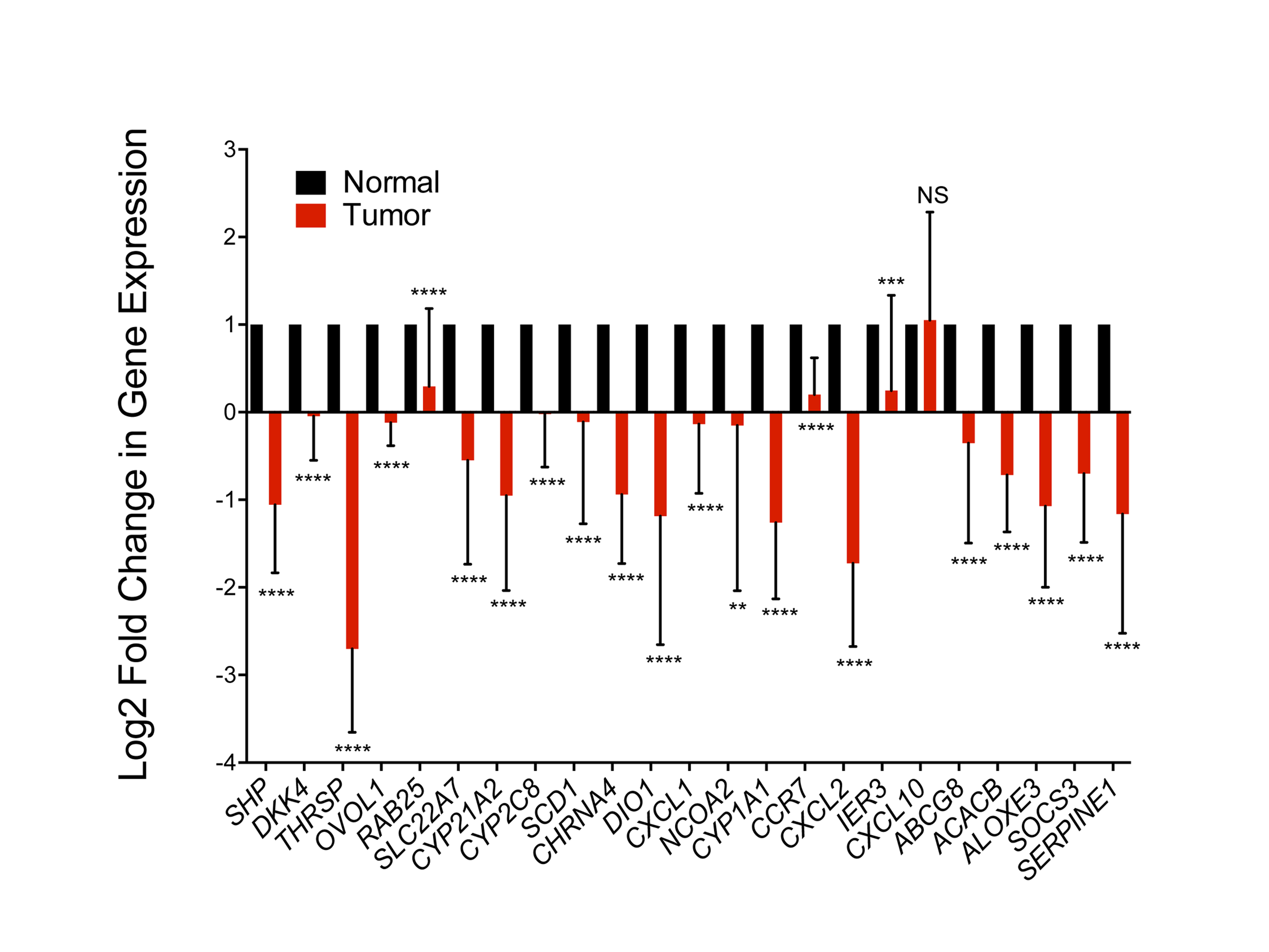

Supplement: S3 Fig — A gene expression profiling dataset (GSE1898) was analyzed using GEO2R to generate individual gene expression profiles for SRC-2 target genes across 91 human HCCs relative to a pooled normal liver reference. Student’s t-test was performed to assess statistical significance, ** = p<0.01; *** = p<0.001; **** = p<0.0001. (TIF) [file pgen.1006650.s007.tif]

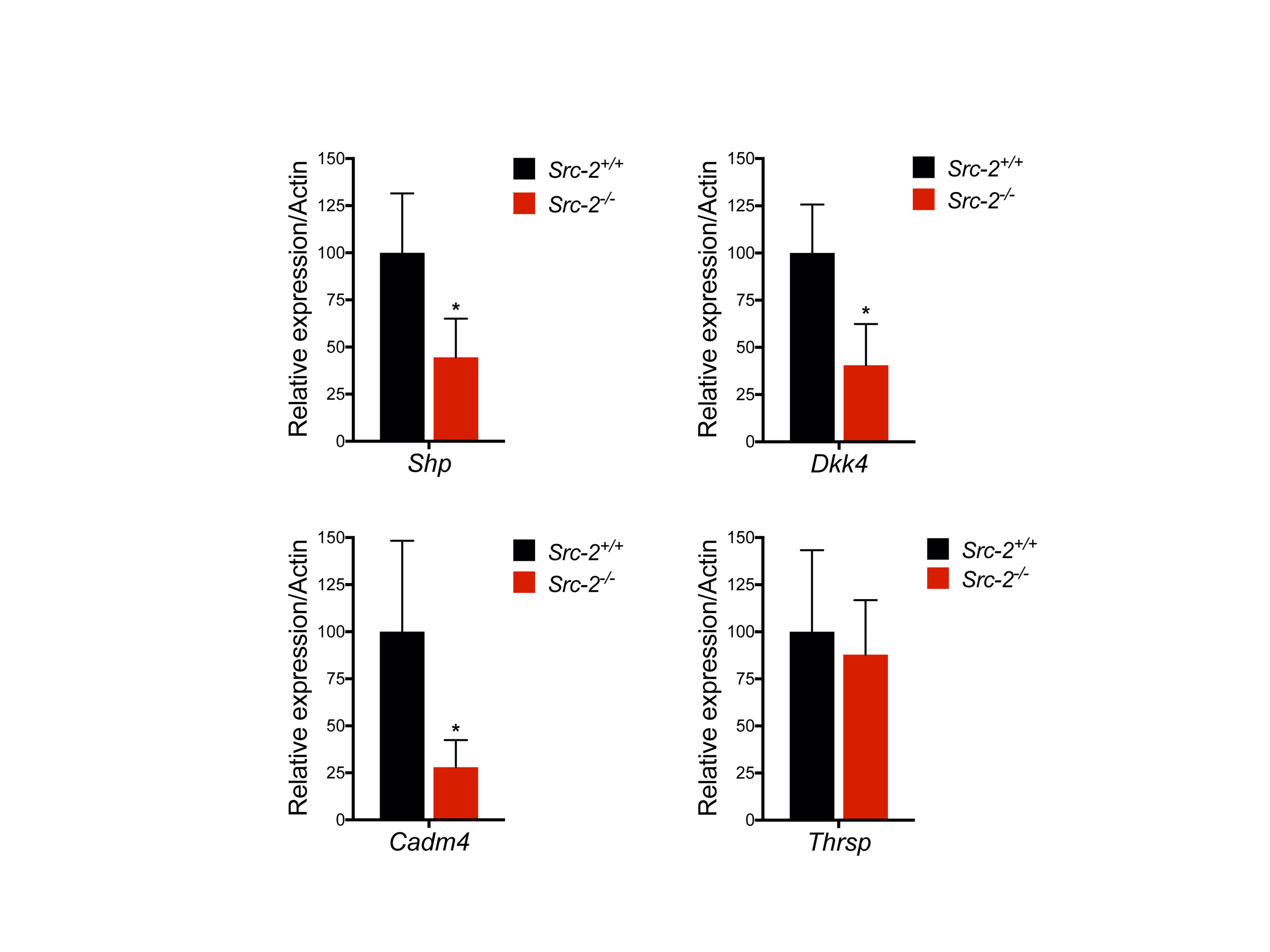

Supplement: S4 Fig — Real-time PCR quantification of Shp, Dkk4, Cadm4, and Thrsp expression in four independent liver tumors from Src-2-/- and Src-2+/+ mice. Bar graphs represent mRNA expression normalized to Actin and error bars represent SDs from triplicate measurements (n = 4 tumors per group). Student’s t-test was performed to assess statistical significance (* = p<0.05). (TIF) [file pgen.1006650.s008.tif]

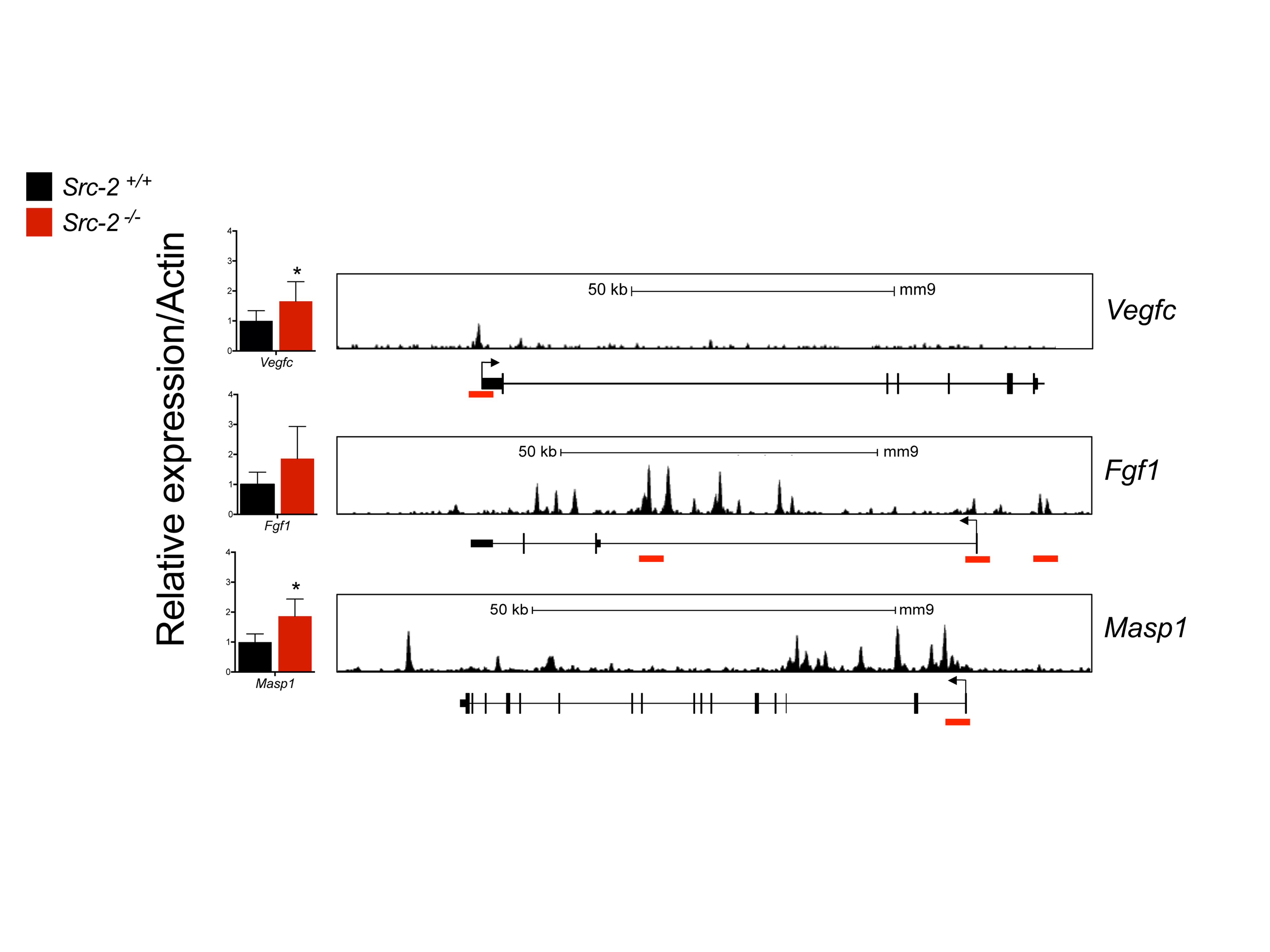

Supplement: S5 Fig — Left, Real-time PCR quantification of Vegfc, Fgf1 and Masp1 in Src2+/+ and Src2-/- liver tumors. Bar graphs represent mRNA expression normalized to ACTIN and error bars represent SDs from triplicate measurements measured in five tumors per group. Student’s t-test was performed to assess statistical significance. Right, mouse liver SRC-2 ChIP-Seq peaks depicting SRC-2 binding sites in promoter regions of Vegfc, Fgf1 and Masp1. (TIF) [file pgen.1006650.s009.tif]

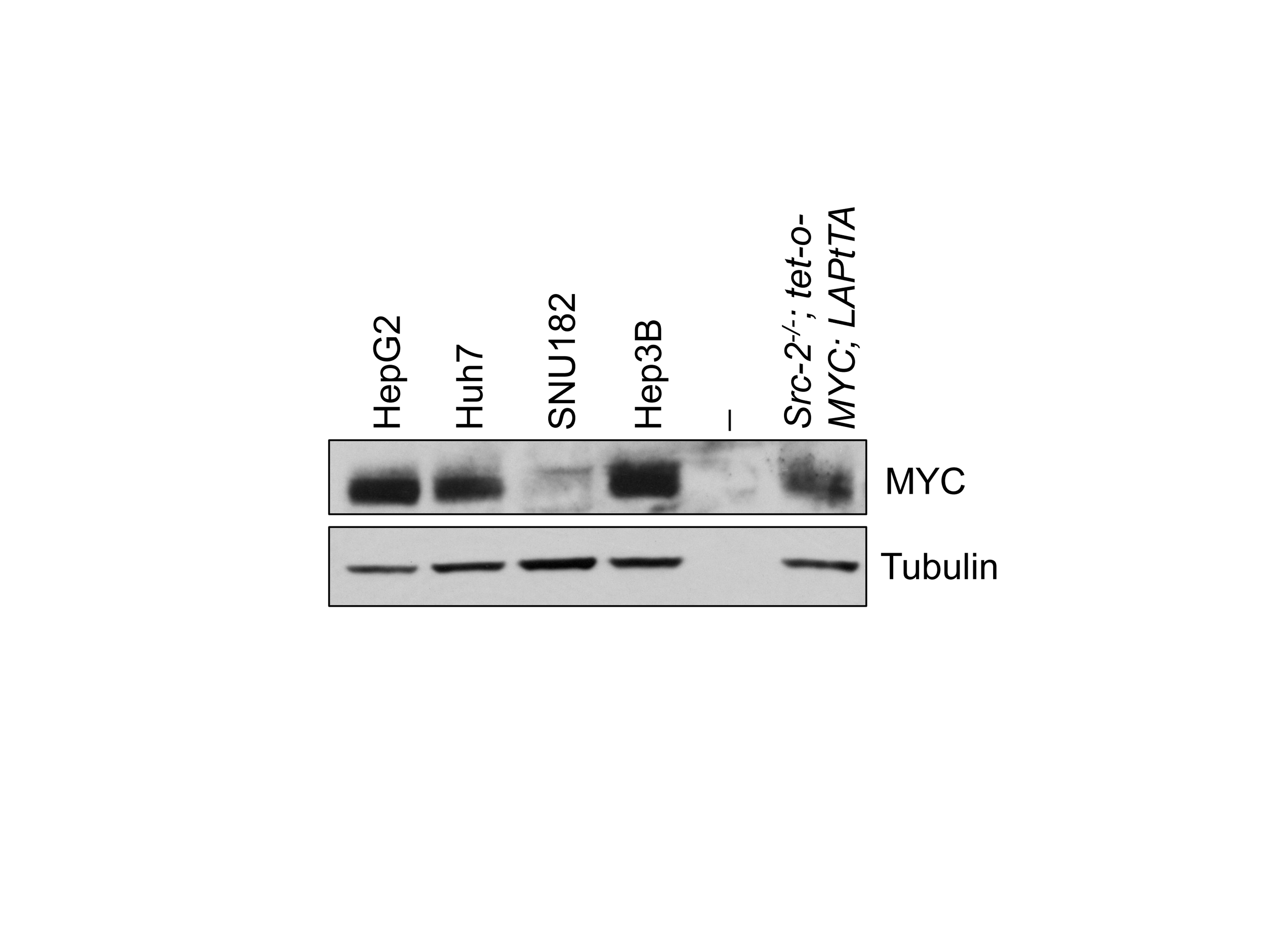

Supplement: S6 Fig — Western blot analysis depicting MYC protein levels in a panel of human liver cancer cells and a liver tumor from an Src-2-/-; tet-o-MYC; LAPtTA animal (after dox removal, with MYC overexpression). Of note, MYC levels were not experimentally modulated in any of the human liver cancer cell lines used in these studies. (TIF) [file pgen.1006650.s010.tif]

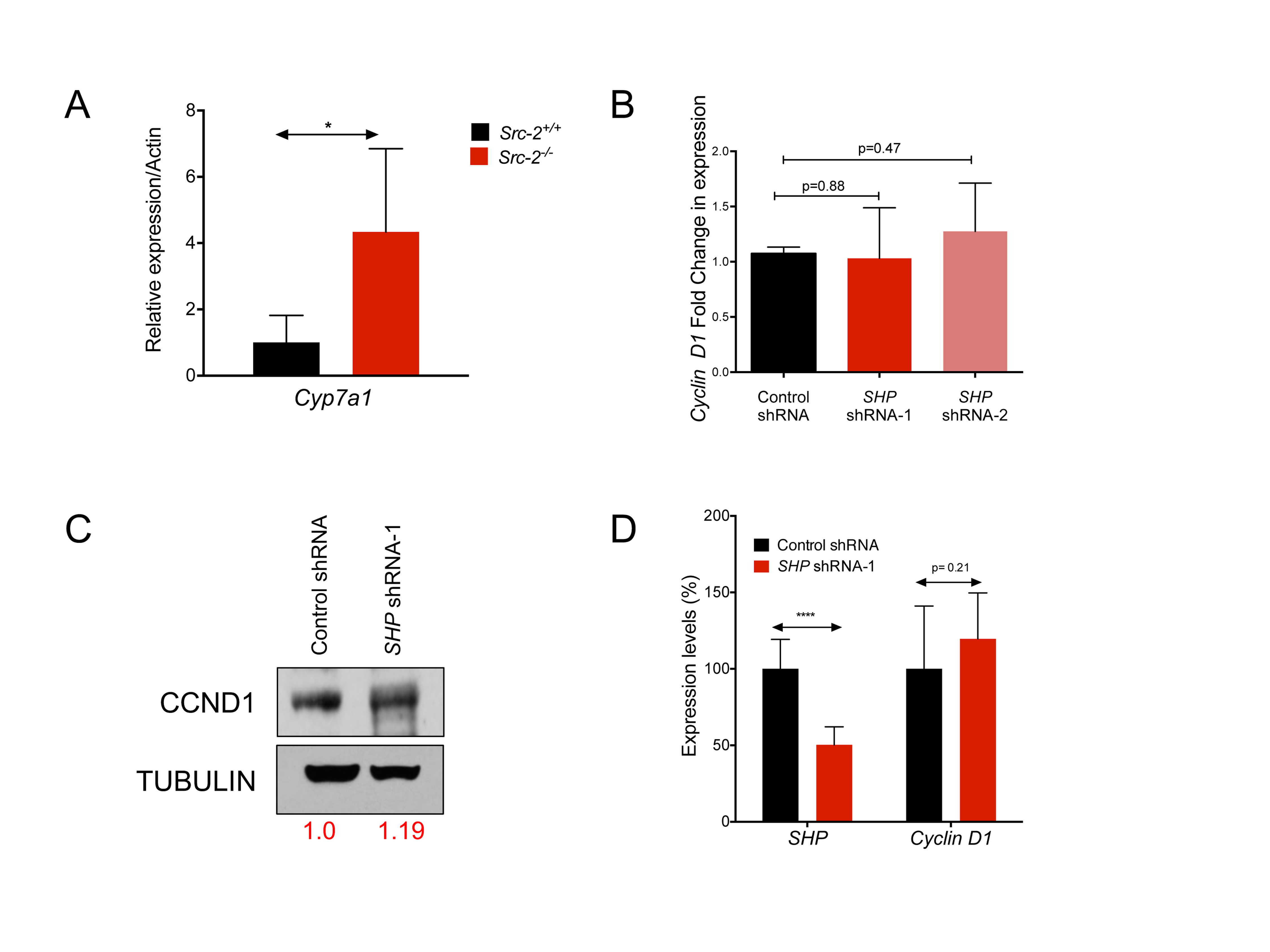

Supplement: S7 Fig — (A) Real-time PCR quantification of Cyp7a1 expression in Src-2+/+ and Src-2-/- liver tumors. Bar graphs represent mRNA expression of Cyp7a1 normalized to ACTIN and error bars represent SDs from triplicate measurements (n = 4 tumors per group). Student’s t-test was performed to assess statistical significance (* = p<0.05). (B) Real-time PCR quantification of CYCLIN D1 expression levels in SHP shRNA Huh7 cells. Bar graphs represent mRNA expression of CYCLIN D1 normalized to ACTIN and error bars represent SDs from triplicate measurements. Student’s t-test was performed to assess statistical significance. (C) Western blot analysis depicting CYCLIN D1 protein levels in SHP shRNA and control shRNA cells. (D) Real-time PCR quantification of CYCLIN D1 expression levels in tumors derived from xenograft assays with control or SHP shRNA-1. Bar graphs represent mRNA expression of CYCLIN D1 normalized to ACTIN and error bars represent SDs from triplicate measurements (n = 4 tumors per group). Student’s t-test was performed to assess statistical significance (**** = p<0.0001). (TIF) [file pgen.1006650.s011.tif]

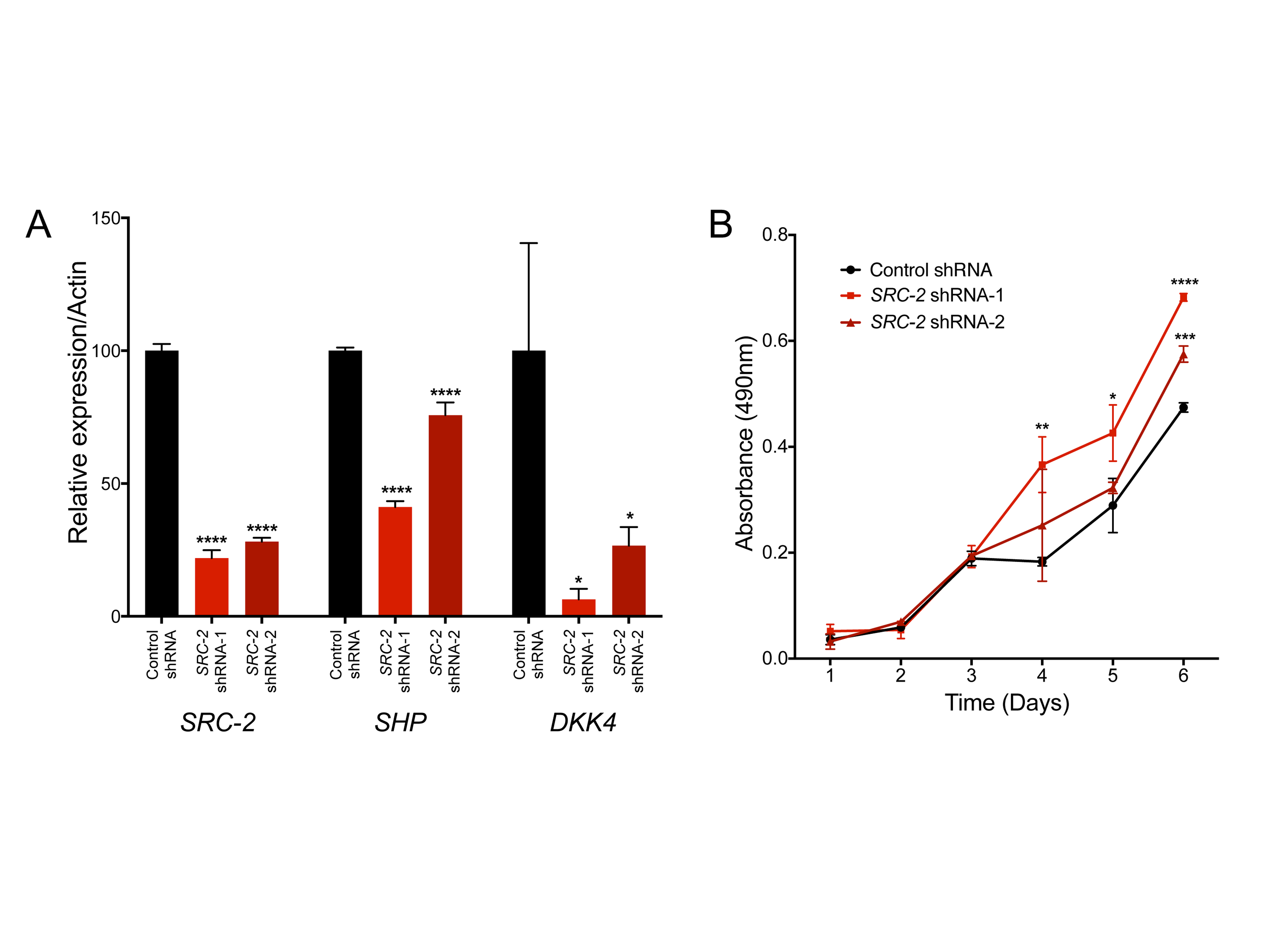

Supplement: S8 Fig — (A) Real-time PCR quantification of SRC-2, SHP, and DKK4 expression in Huh7 cells after inhibition of SRC-2 with two independent shRNAs. Bar graphs represent mRNA expression of the labeled transcript normalized to ACTIN and error bars represent SDs from triplicate measurements. (B) MTS assay measuring proliferation of Huh7 cells with control shRNA, SRC-2 shRNA-1, or SRC-2 shRNA-2. Error bars in real-time quantitation and proliferation assays represent SDs from triplicate measurements. A student’s t-test was performed to determine statistical significance. * = p<0.05; ** = p<0.01; *** = p<0.001; **** = p<0.0001. (TIF) [file pgen.1006650.s012.tif]

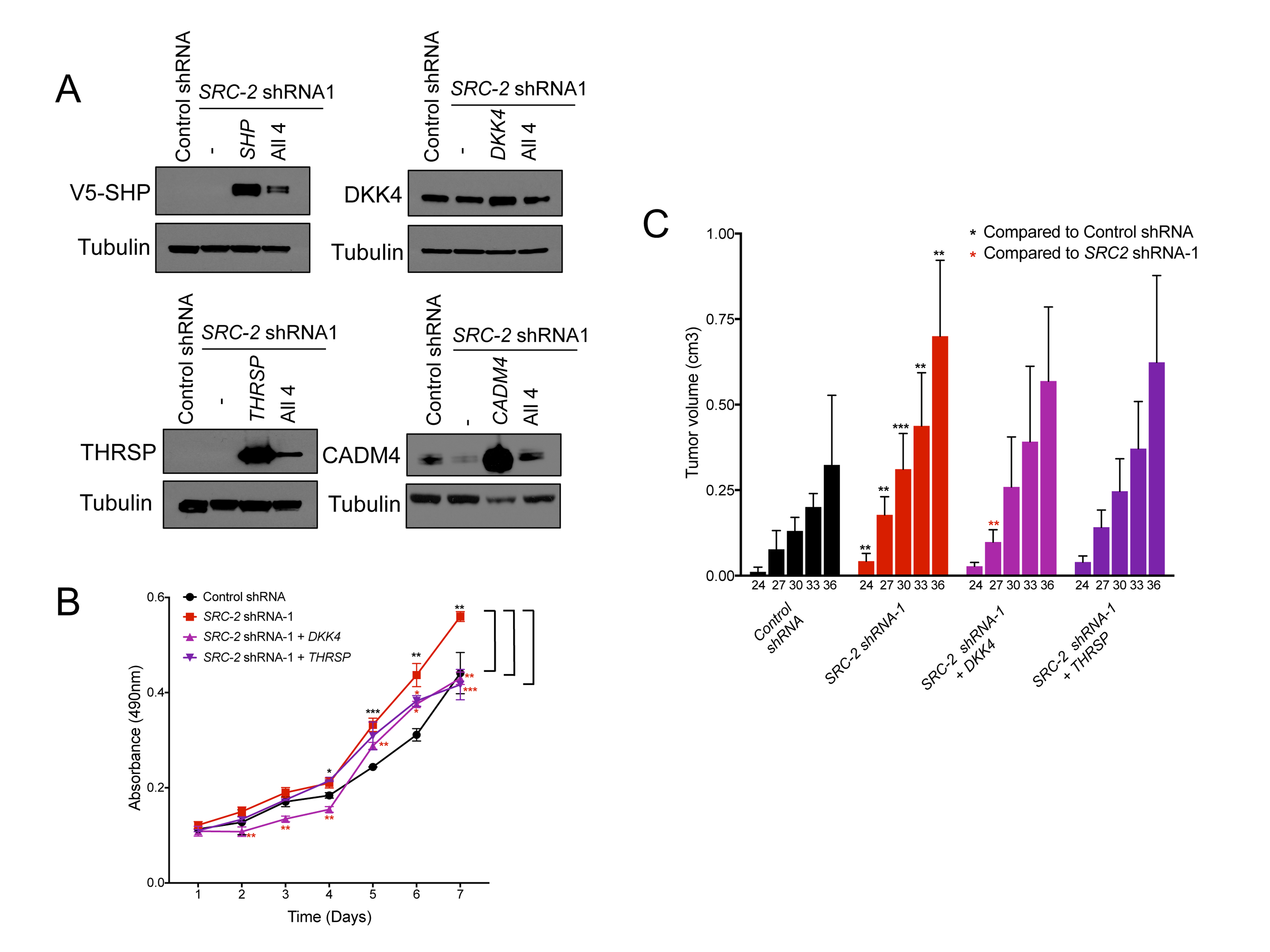

Supplement: S9 Fig — (A) Western blot analysis demonstrating inhibition of SRC-2 targets in HepG2 cells expressing SRC-2 shRNA-1, as compared to control shRNA cells, and overexpression of each of the four targets alone or in combination in SRC-2 shRNA-1 cells. The V5 antibody detects V5-tagged SHP in the rescue experiment, but does not recognize endogenous SHP in HepG2 cells. (B) MTS assay measuring proliferation of HepG2 cells with control shRNA, SRC-2 shRNA-1, or SRC-2 shRNA-1 with overexpression of THRSP or DKK4 alone. (C) Quantification of tumor volumes in nude mice injected with HepG2 cells as described in (B). Bars represent mean tumor volumes. Error bars in proliferation assays represent SDs from triplicate measurements. Error bars in xenograft experiments represent SDs from a total of ten subcutaneous injections (n = 5 mice) per shRNA tested. A student’s t-test was performed to determine statistical significance. * = p<0.05; ** = p<0.01; *** = p<0.001. Black asterisks represent comparisons to the control shRNA. Red asterisks represent comparisons to SRC-2 shRNA-1. (TIF) [file pgen.1006650.s013.tif]

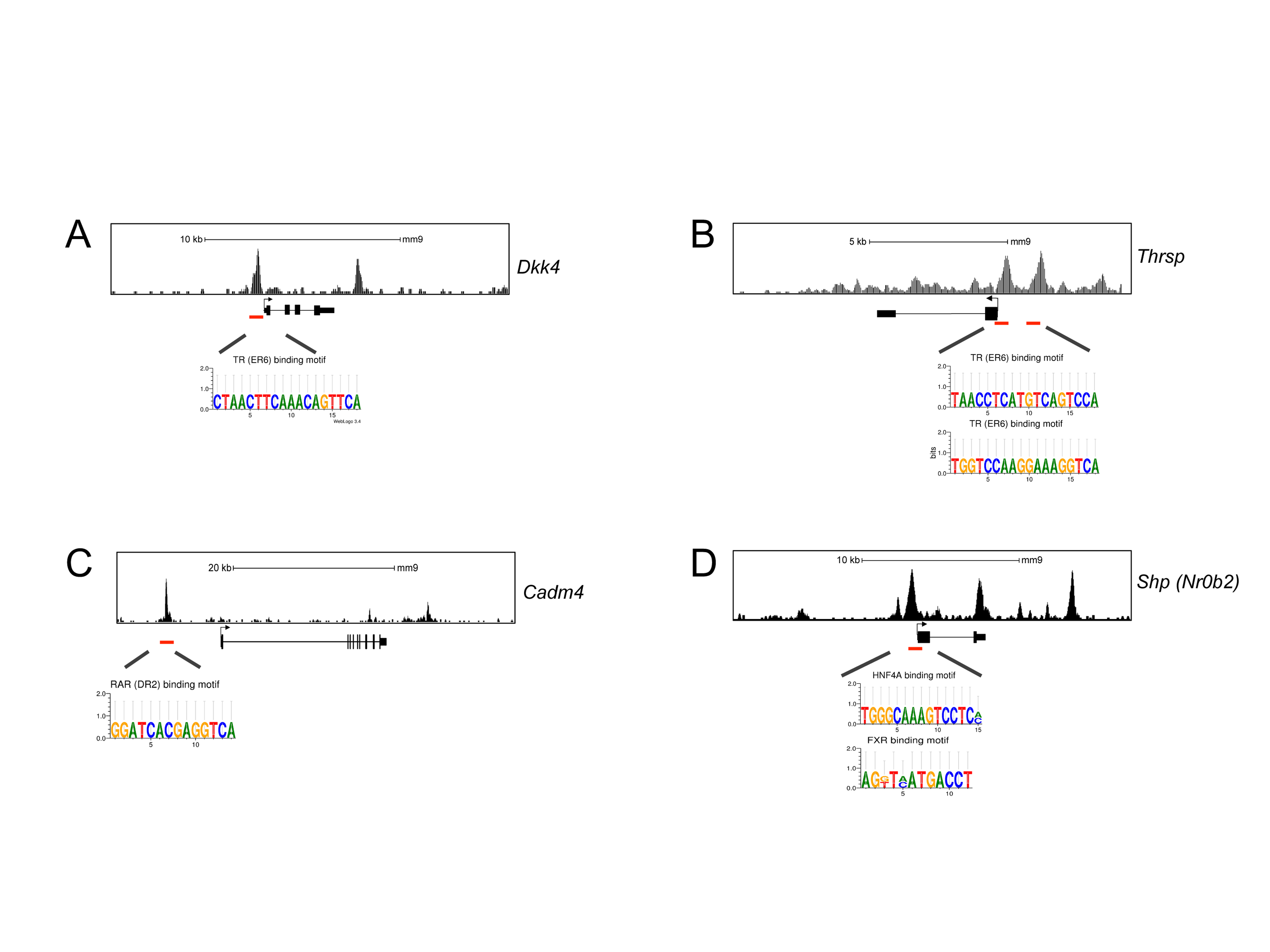

Supplement: S10 Fig — Depiction of NR binding motifs in the promoter regions of Dkk4 (A),Thrsp (B), Cadm4 (C), and Shp (D) as predicted by NHRscan. SRC-2 ChIP-Seq peaks for Shp, Dkk4, Thrsp, and Cadm4 are depicted with SRC-2 binding sites represented by red bars as well as nucleotide consensus sequences corresponding to putative NR binding motifs. (TIF) [file pgen.1006650.s014.tif]

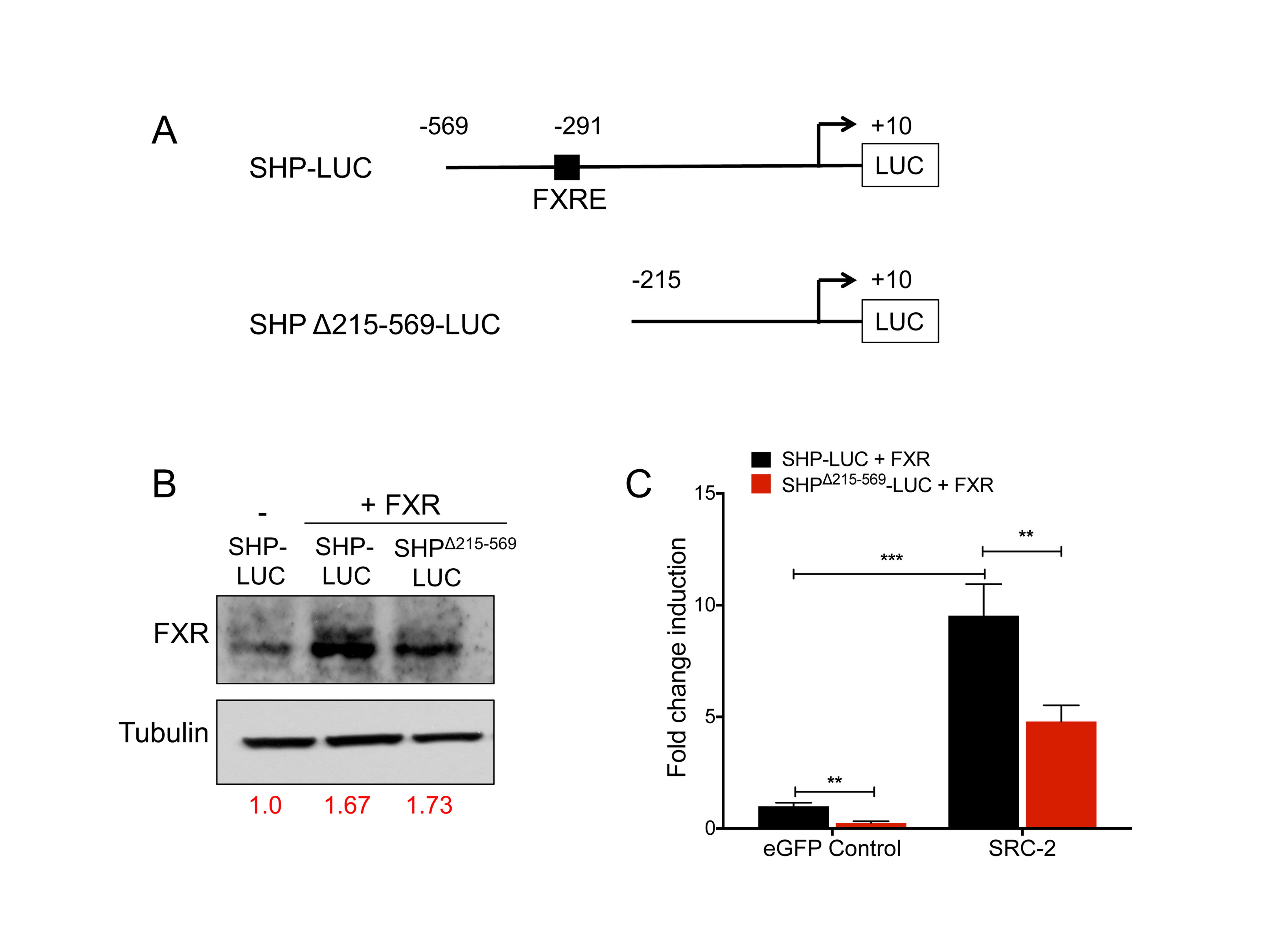

Supplement: S11 Fig — (A) eGFP control or SRC-2 expressing Huh7 cells were transfected with FXR plasmid (20ng) in combination with SHP-LUC or SHPΔ215-569-LUC reporter plasmids (80ng), 1 ng Renilla control reporter plasmid and 199ng pUC19 plasmid yielding a total of 300 ng DNA per well, and then measured for luciferase activity after 48 hours. (B) Western blot demonstrating expression of FXR in Huh7 cells transfected with SHP-LUC plasmids with and without FXR. Numbers in red represent quantification of FXR protein levels relative to the SHP-LUC only sample and normalized to Tubulin. (C) Quantification of SHP-luciferase fold-induction in eGFP control and SRC-2 expressing cells. (TIF) [file pgen.1006650.s015.tif]

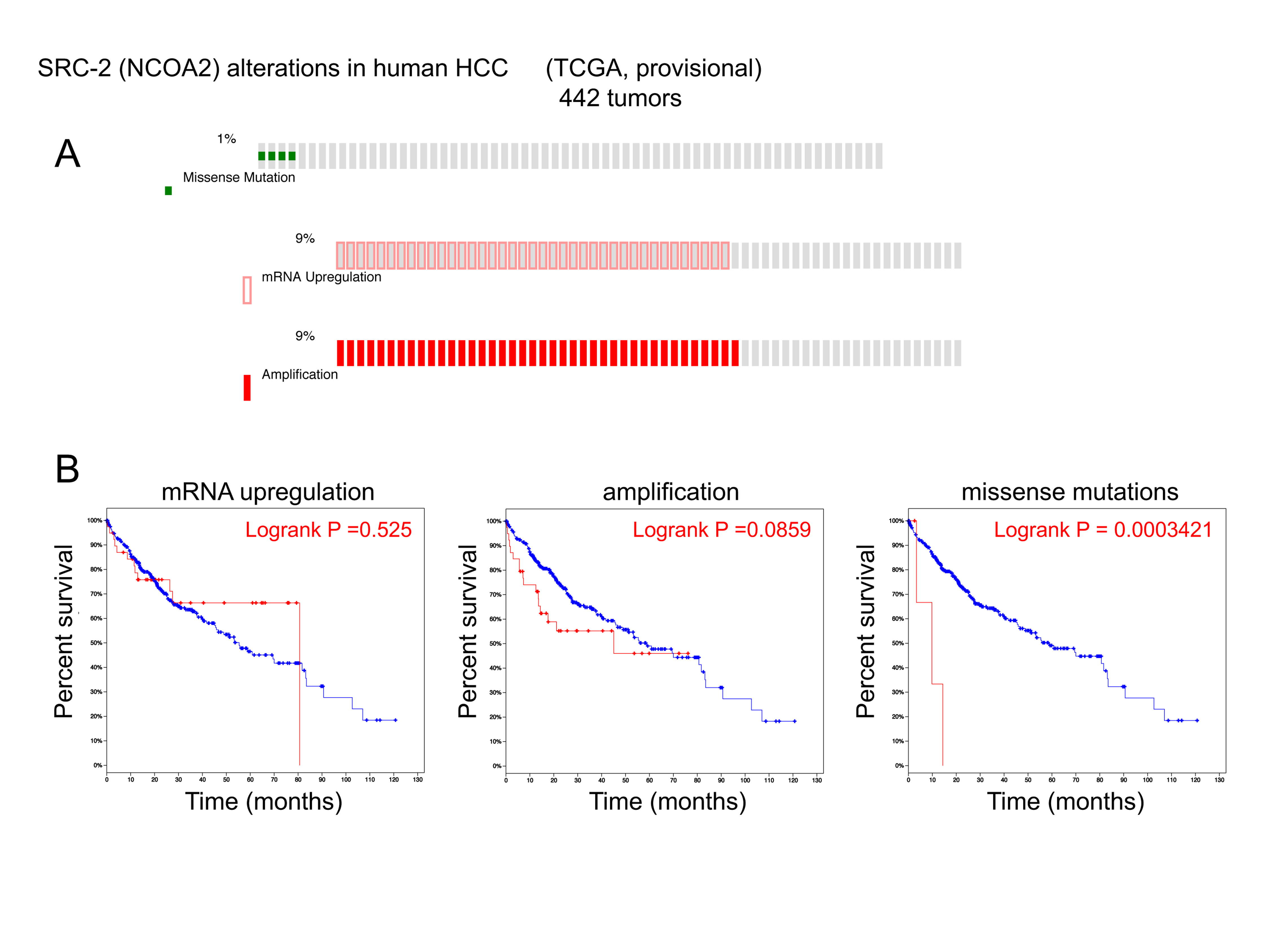

Supplement: S12 Fig — (A) Oncoprints demonstrating mRNA upregulation, amplification, and missense mutations of SRC-2 in 442 HCC tumors from the provisional TCGA dataset. (B) Kaplan-Meier survival analysis of HCC patients with missense mutations, mRNA upregulation, and amplification of SRC-2. (TIF) [file pgen.1006650.s016.tif]
